# Overcoming Resistance to Natural Killer Cell Based Immunotherapies for Solid Tumors

**DOI:** 10.3389/fonc.2019.00051

**Published:** 2019-02-11

**Authors:** Gaurav Nayyar, Yaya Chu, Mitchell S. Cairo

**Affiliations:** ^1^Department of Pediatrics, New York Medical College, Valhalla, NY, United States; ^2^Department of Cell Biology & Anatomy, New York Medical College, Valhalla, NY, United States; ^3^Department of Microbiology & Immunology, New York Medical College, Valhalla, NY, United States; ^4^Department of Medicine, New York Medical College, Valhalla, NY, United States; ^5^Department of Pathology, New York Medical College, Valhalla, NY, United States

**Keywords:** natural killer cell, chimeric antigen receptor, immunotherapy, solid tumor, cytokines, tumor microenvironment, checkpoint inhibitors, bispecific antibody

## Abstract

Despite advances in the diagnostic and therapeutic modalities, the prognosis of several solid tumor malignancies remains poor. Different factors associated with solid tumors including a varied genetic signature, complex molecular signaling pathways, defective cross talk between the tumor cells and immune cells, hypoxic and immunosuppressive effects of tumor microenvironment result in a treatment resistant and metastatic phenotype. Over the past several years, immunotherapy has emerged as an attractive therapeutic option against multiple malignancies. The unique ability of natural killer (NK) cells to target cancer cells without antigen specificity makes them an ideal candidate for use against solid tumors. However, the outcomes of adoptive NK cell infusions into patients with solid tumors have been disappointing. Extensive studies have been done to investigate different strategies to improve the NK cell function, trafficking and tumor targeting. Use of cytokines and cytokine analogs has been well described and utilized to enhance the proliferation, stimulation and persistence of NK cells. Other techniques like blocking the human leukocyte antigen-killer cell receptors (KIR) interactions with anti-KIR monoclonal antibodies, preventing CD16 receptor shedding, increasing the expression of activating NK cell receptors like NKG2D, and use of immunocytokines and immune checkpoint inhibitors can enhance NK cell mediated cytotoxicity. Using genetically modified NK cells with chimeric antigen receptors and bispecific and trispecific NK cell engagers, NK cells can be effectively redirected to the tumor cells improving their cytotoxic potential. In this review, we have described these strategies and highlighted the need to further optimize these strategies to improve the clinical outcome of NK cell based immunotherapy against solid tumors.

## Introduction

Natural Killer (NK) cells are the effector cells that constitute a key part of the innate immune system. They have emerged as a promising option for immunotherapy of a variety of malignancies due to their ability to identify and kill cancer cells without any prior sensitization. NK cells have unique ability to differentiate between the normal and transformed cells. They possess a variety of activating and inhibitory receptors, and their net functional outcome is a complex integration of signals between these activating and inhibitory receptors. Over the past few decades, significant advances have been made in successfully targeting hematologic malignancies with the use of novel immunotherapeutic strategies. However, solid tumors continue to pose unique therapeutic challenges, and the conventional cytoreductive therapies have proven to be of limited efficacy. NK cell based therapeutic strategies have been applied against solid tumor with only modest success. The ability of solid tumor cells to escape the immune-surveillance, proliferate rapidly and metastasize when coupled with the abnormalities in the NK cells like decreased expression of activating receptors or overexpression of inhibitory receptors, decreased activation and persistence, defective cytokine production, abnormal intracellular signaling molecules, inefficient trafficking to the tumor site, and senescence resulting in a defective cytolytic response are likely the major contributors to the poor response of NK cells based strategies against solid tumors. In this review, we have attempted to address the unique characteristics of solid tumors and their microenvironment, mechanisms contributing to the NK cell resistance and describe the various applications that could be applied in an attempt to enhance the therapeutic potential of NK cells against solid tumors.

## Challenges in Treating Solid Tumors

NK cell based immunotherapies have been used widely and successfully for different hematologic malignancies, particularly acute myeloid leukemia. One of the early studies in patients with relapsed acute myeloid leukemia, showed haploidentical NK cell infusion in combination with high dose fludarbine and cyclophosphamide caused expansion of donor NK cells, significantly increased endogenous Interleukin (IL)-15 and achieved a complete hematologic remission in 5 of 19 (26%) patients (1). More recently, NK cell based therapies have emerged as an attractive strategy for targeting solid tumors. However, there are some considerable challenges in use of NK cell based therapies against solid tumors. Solid tumors are a very heterogeneous group of malignancies that have historically been more difficult to treat even with the use of multimodal approaches. This heterogeneity could be due to differences in evolution of these tumors caused by varying gene profile signature, different mutations and involvement of different cell signaling pathways (2, 3). One of the major challenges with NK cell based therapies against solid tumors is the trafficking of these immune cells to the tumor location and infiltration into the tumor. Multiple studies have shown that the tumor progression and outcomes correlate with the presence of NK cells at the tumor site (4–6). The density of NK cells infiltrating into the tumor has been shown to be an independent predictor of the progression free survival in gastrointestinal stromal tumors, and in pulmonary adenocarcinoma (4, 7). The chemokines expressed on the surface of NK cells, and the ones secreted by the tumor cells play a central role in NK cell infiltration into the tumor (8).

It has been well established that tumor microenvironment plays a key role in the proliferation and survival of the cancerous cells. Tumor microenvironment consists of a variety of cells including tumor associated fibroblasts, tumor associated macrophages, dendritic cells, neutrophils, regulatory T cells (Tregs), myeloid derived suppressor cells (9), that provide a constant chronic inflammatory milieu leading to angiogenesis, tumor cell survival and proliferation. The presence of inhibitory signals in the tumor microenvironment and altered immunogenicity of tumor cells also leads to poor infiltration and activation of NK cells into the tumor. Furthermore, rapidly growing solid tumors create an environment of localized hypoxia (10). The low oxygen tension in the solid tumor tissue not only creates metabolic disturbances in the tumor microenvironment but also leads to generation of reactive oxygen species. This cellular environment of hypoxia is mediated by a variety of transcriptional regulators primarily, hypoxia inducible factor-1 (11). Poorly oxygenated tumor cells undergo adaptive changes at the proteomic level leading to transcriptional activity resulting in inhibition of apoptosis and promoting angiogenesis and upregulation of the tumor growth factors (12). Net result is the continued survival and proliferation of the tumor cells with an aggressive phenotype, that frequently metastasize to distant tissues, and are relatively resistance to treatment (11).

Recently, a lot of advances have been made in targeting hematologic malignancies using novel immunotherapeutic strategies like chimeric antigen receptors (CAR). However, the success stories have been less exciting against solid tumors, particularly due to lack of appropriate immunologic targets, that are highly expressed on surface of tumor tissue with relative absence on the non-vital tissues to avoid “on-target/off-tumor” effects (13). In addition, these genetically modified effector cells have to overcome the challenges posed by the physical barriers preventing infiltration into the tumor tissues and hostile tumor microenvironment (14, 15). Antigen escape phenomenon due to downregulation or loss of targetable antigen happens frequently in solid tumors rendering these CAR based therapies less effective (14, 16). However, unlike CAR T cells, NK cell based therapies have the advantage of overcoming the limitation posed by the antigen escape mechanism to a certain extent due to their inherent ability to recognize and kill tumor cells without prior sensitization. Furthermore, NK cell alloreactivity following haploidentical SCT is protective against graft vs. host disease while producing a robust graft vs. tumor/leukemia effect (17, 18).

## Natural Killer Cell Biology and Target Recognition

Natural killer cells represent human body's first line of defense against tumor cells and infectious pathogens and play a key role in tumor immune surveillance. NK cells were initially identified in mice when investigators noticed a large granular subtype of lymphocytes distinct from T and B lymphocytes, and possessed cytotoxic activity against mouse tumor cell lines (19, 20). Phenotypically, NK cells lack B and T cells markers CD19/TCR/CD3 on their cell surface but they express CD16 and CD56 surface antigens. NK cells are further characterized by the degree of CD56 expression into dim and bright subsets where the subtypes have significant differences in terms of cytokine production, response to cytokines and their killing potential. Around 90% of the NK cells, including the alloreactive NK cells, express low levels of CD56 but have high expression of CD16 (CD16^bright^ CD56^dim^), and are generally found in the peripheral circulation. These cells are considered to be the “mature” NK cells and have higher cytotoxic potential. The remaining 10% of the NK cells are CD16^dim^CD56^bright^ with higher levels of CD56 expression, and they are considered “immature” NK cells (21, 22). These immature or unlicensed NK cells generally reside in the lymphoid tissues but they are more responsive to stimulation and respond readily by secreting a variety of cytokines including interferon γ (IFN-γ), tumor necrosis factor (TNF)-α, IL-5, IL-10, and IL-13 (4, 23). All subsets of NK cells express intermediate affinity heterodimeric IL-2 receptor. However, high affinity receptors and c-kit tyrosine kinase is only expressed by CD56^bright^ NK cells, which gives them the unique ability to proliferate when exposed to very small concentrations of IL-2 (24, 25). In addition, there is also a differential expression of adhesion molecules between the two NK cell subsets. CD56^bright^ cells have higher levels of expression of chemokine receptor type-7 and L-selectin which likely helps these cells to traffic to secondary lymphoid organs, whereas CD56^dim^ cells have a higher level of expression of Leucocyte function-associated antigen-1, providing them the unique migratory properties in response to foreign pathogens (26). Therefore, CD56^dim^ NK cells appear to have a predominantly cytotoxic function naturally, and CD56^bright^ cells play a more immunomodulatory role. However, it is still unclear if these subsets represent just different stages in maturation of NK cells or if they are completely different cells emerging from a common hematopoietic precursor (3). Besides the circulation system, distinct subsets of NK cells also reside in tissues and organs (22). NK cells in lymph nodes, tonsils and spleen differ from NK cell subsets in peripheral blood by phenotypes and functions (27, 28).

NK cells do not require prior sensitization to target the transformed cells (4). NK cell receptor can play a stimulatory or an inhibitory role and has the unique ability to recognize major histocompatibility complex−1 (MHC-1) or MHC-1 like molecules on the target cells. The balance between the inhibitory signals received from the killer inhibitory receptors and natural killer group protein 2 family member A (NKG2A) and killer cell lectin-like receptor subfamily G member 1; and the stimulatory receptors including natural cytotoxicity receptors, NKp30, NKp44, NKp46, natural killer group protein 2 family member D (NKG2D) defines the net functional outcome of the NK cells.

**Figure 1:**
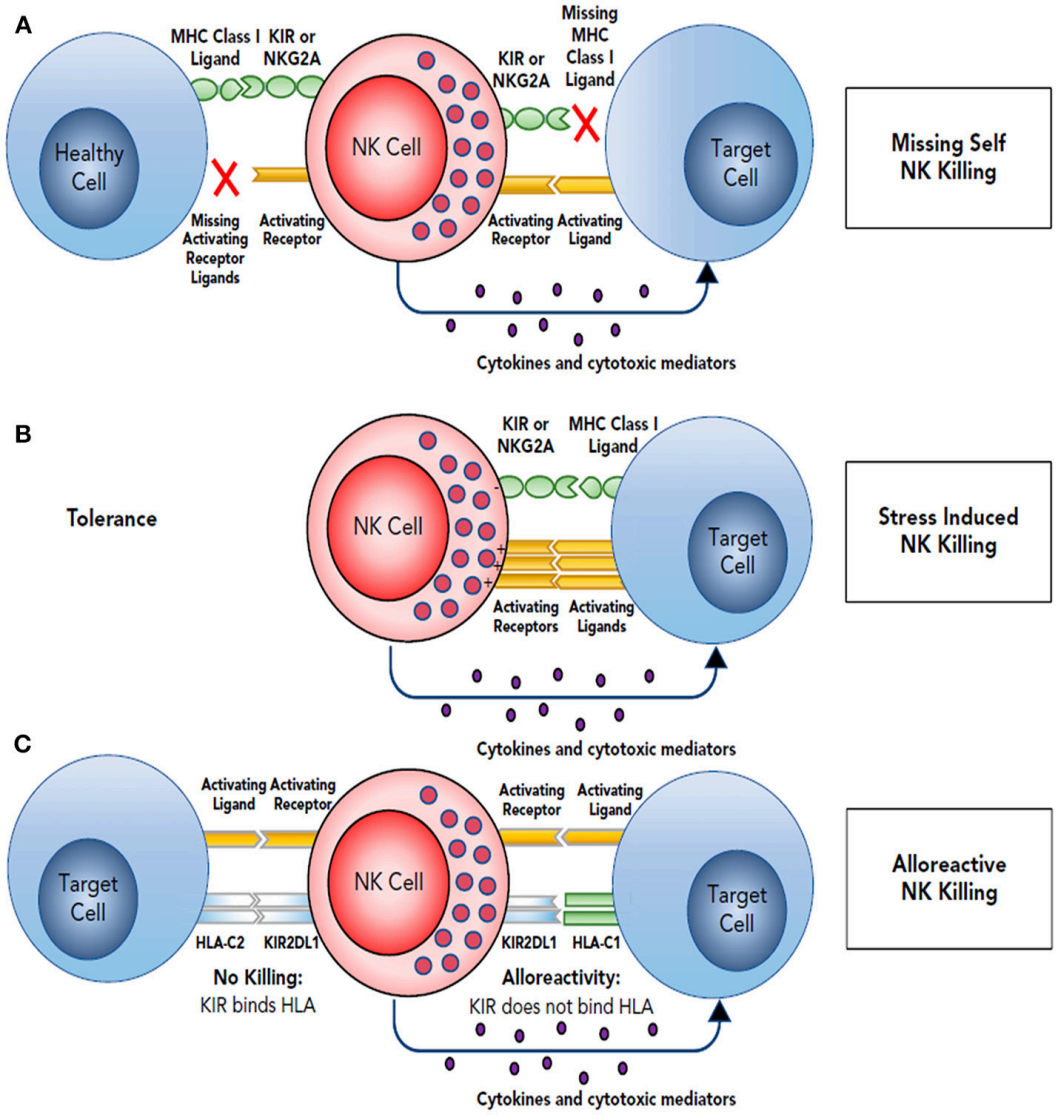
Target recognition, tolerance, missing self. **(A)** NK cells recognize and kill their targets by an integrated balance of inhibitory and activating signals to discriminate between healthy cells (tolerance) vs. elimination of transformed or virally infected targets (killing). NK-cell tolerance depends on several MHC class I inhibitory signals (either classical, HLA-A, -B, or -C, or nonclassical, HLA-E) expressed by healthy cells that engage KIR or NKG2A with minimal activation signals resulting in tolerance. Malignant transformation or viral infection promotes target cell killing by downregulation of MHC class I expression and an upregulation of signals from activating NK-cell receptors. **(B)** Although in some cases, MHC downregulation is variable or incomplete, target cell killing can still occur by changing the balance with activating signals upregulated by stress-induced activating receptor ligands. **(C)** This balance between inhibition and activation can be uniquely manipulated in the hematopoietic transplant setting by selection of donors who will respond to apparent missing self HLA class I in the HLA-mismatched recipients. For example, reconstitution with a high frequency of donor KIR2DL1^+^ NK cells would not be inhibited in a HLA-C1 (C1^+^-HLA-C) recipient (KIR ligand mismatch). Here, NK-cell alloreactivity would kill the recipient's tumor. In contrast, when the same KIR2DL1^+^ NK cells reconstitute in an HLA-C2 recipient (C2^+^-HLA-C) (KIR ligand match), the recipient's tumor would be seen as having self HLA class I and would not provoke an alloreactive NK-cell response. Reproduced with permission from Cooley et al. (30).

NK cells recognize autologous cells that express human leucocyte antigen (HLA) Class I molecules that prevent them from attacking the host tissue, known as “tolerance to self.” During viral infections or malignant transformation, there is decreased expression of MHC class I antigens on cell surface in order to avoid recognition by the antitumor T cells. NK cells that are surveilling the tissues for a normal level of MHC class I expression, recognize this as “altered self” resulting in decreased engagement of the killer inhibitory receptors and increase expression of the stimulatory receptors resulting in effector response and cytotoxic killing of the transformed cells (29). The mechanism of target recognition by NK cells is depicted in Figure 1. There are several mechanisms by which NK cells can kill the target cells without any prior sensitization. They can exert direct cytotoxicity through release of granules containing perforin and granzyme (31). NK cells also have the unique ability exert antibody-dependent cell mediated cytotoxicity (ADCC) due to presence of Fc receptor FcγRIIIa that recognizes the Fc portion of the antibodies. In addition, they can mediate cytotoxicity via apoptotic pathways involving fas ligand or TNF-related apoptosis-inducing ligand (32, 33).

## NK Resistance Mechanisms

NK cells have shown significant alloreactive anti-leukemic effects against liquid tumor cells especially following haploidentical SCT (stem cell transplantation) (17) and the higher NK cell immune reconstitution in the early post allogeneic SCT period has also been demonstrated to be associated with significantly improved survival and lower leukemia relapse rates (34, 35). However, the adoptive transfer of autologous NK cells showed no clinical response in patients with progressive stage IV melanoma or renal cell carcinoma (RCC) (36). In solid tumor clinical trials, NK cells often display impaired functions in patients and impaired NK cell-function is related to high disease stages and poor prognosis (37, 38). The NK resistance, on NK side, is mainly due to the small numbers of active NK cells, the short lifespan of NK cells, poor persistence and trafficking, and lack of specific tumor targeting (39). On the tumor side, tumor cells make up a microenvironment that inhibits NK cell activity by altering the balance between NK activating and inhibitory receptors such as reducing NK activating receptor NKG2D and CD16, secreting inhibitory factors such as transforming growth factor beta (TGF-β), IL-6 and IL-10, shedding NKG2D ligands such as MHC class I chain-related protein A (MICA) and MHC class I chain-related protein B (MICB), and recruiting suppressive immune cells such as Tregs and myeloid derived suppressor cells (40). Table 1 summarizes the potential mechanisms of resistance to NK cell based therapy of solid tumors.

**Table 1:** NK cell resistance mechanisms against solid tumors.

**Mechanism**	**References**
Abnormal NK cell trafficking to the tumor location	(4, 41)
Poor NK cell infiltration into the tumor	(6–8)
Abnormal NK cell function/activity in patients with malignancy	(37)
Lack of unique targetable tumor antigens	(13, 42)
Antigen escape/downregulation following targeted therapies	(16)
Increased angiogenesis and upregulation of tumor growth factors by hypoxic microenvironment	(11, 12)
Hypoxia induced shedding of MHC class I chain-related (MIC) on tumor cells and downregulation of NKG2D on effector cells	(43)
Chronic immunosuppressive signals in the tumor microenvironment inhibiting NK cell function and activity	(4)

## NK Resources for Adoptive Therapy

**Figure 2:**
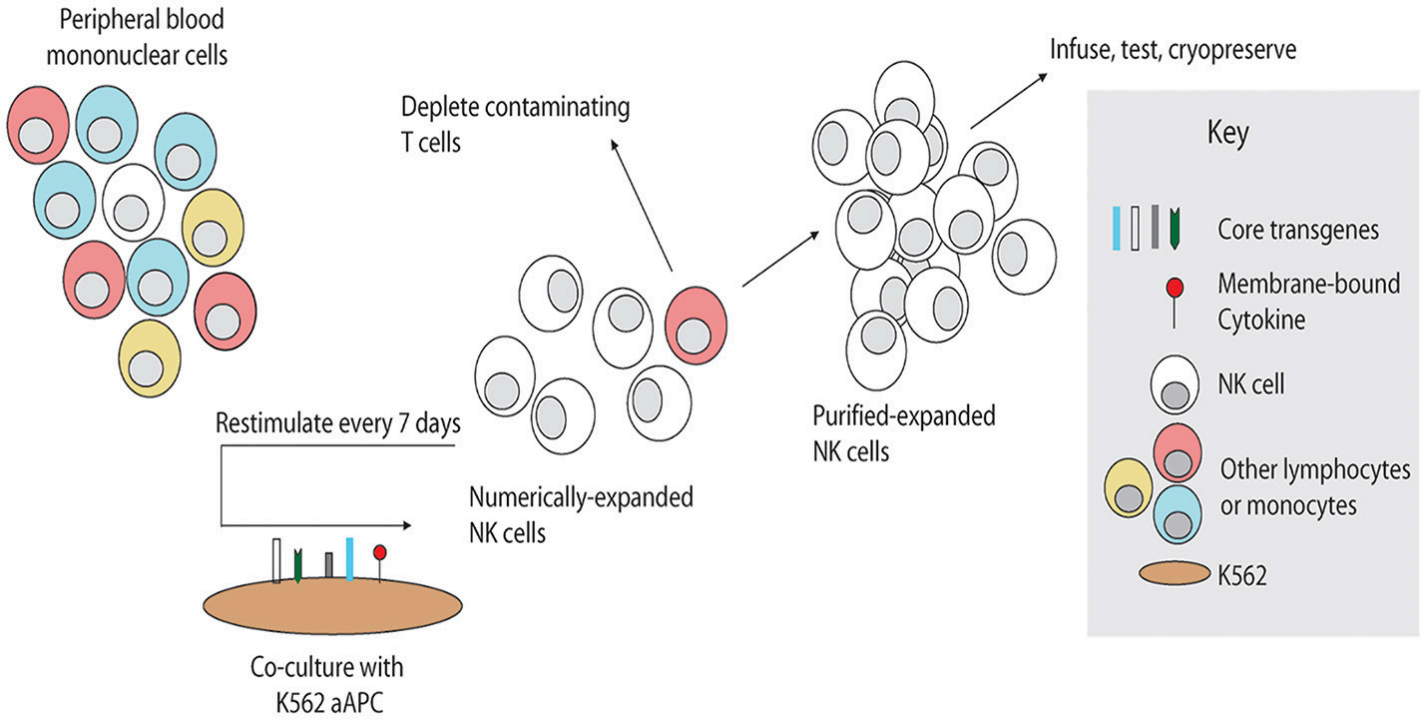
Schema for NK cell manufacturing with artificial antigen-presenting cells. Artificial antigen-presenting cells were produced by genetic modification of K562 to express costimulatory molecules and membrane-bound cytokines. To expand NK cells *ex vivo*, unfractionated PBMC are stimulated weekly with irradiated PBMC, inducing rapid proliferation of NK cells and in some cases non-specific expansion of T cells. Contaminating T cells may be depleted, and the remaining purified NK cells may be stimulated weekly by the artificial antigen-presenting cells as needed to obtain sufficient numbers. Expanded NK cells may be used directly or cryopreserved for future use. Reproduced with permission from Denman et al. (49).

Four sources of active NK cells for adoptive transfer have been reported: autologous NK cells, allogeneic NK cells from donors, NK cell lines and embryo stem cell-derived/induced pluripotent stem cells -derived NK cells. Rosenberg et al. evaluated the efficacy of adoptively transferred IL-2 *ex vivo* activated autologous NK cells to patients with metastatic renal carcinoma and melanomas (36). Even the adoptively transferred NK cells persisted for long time, no significant clinical benefit was observed (36), indicating the limitation of utilizing patients' autologous NK cells alone as a therapeutic strategy. Due to the KIR mismatch to kill tumor cells, the adoptive transfer of allogeneic NK cells may have a superior antitumor effect compared with the approaches utilizing autologous NK cells (44). To overcome the limitation of small number of active NK cells in peripheral blood, our group and others have successfully expanded active NK cells *in vitro* by short term culture with cytokines alone, using cytokines and co-culture with irradiated Epstein-Barr virus-transformed lymphoblastoid cell lines as feeder cells, or cytokines and co-culture with K562 cells expressing transfected cell-membrane bound IL-15 and 4-1BBL (45–48). Lee and colleagues have developed a novel method of *ex-vivo* expansion of NK cells by stimulating peripheral blood mononuclear cells (PBMC) with a genetically-engineered feeder cell line, K562-mbIL21-41BBL, resulting in over 35,000-fold increase in NK cells and significant increase in NK cell functional activation (Figure 2) (49). Recently, Lee et al. used an anti-CD16 monoclonal antibody (mAb) for potent activation of resting NK cells and irradiated autologous PBMC (upregulated NKG2D ligand and CD48) for providing a suitable environment (activating receptor-ligand interactions and soluble growth factors) instead of cancer cell-based feeder cells for large-scale expansion of highly purified cytotoxic NK cells (50). These expanded NK cells showed potent cytotoxicity against various cancer cells *in vitro* and efficiently controlled cancer progression in severe combined immunodeficiency mouse models of human colon and lung cancer (50). Allogeneic expanded NK cells, which were expanded using CD3+ T-cell–depletion PBMCs from healthy donors with irradiated autologous PBMCs, mAb to CD3, and 500 IU/mL of IL2, were evaluated in a phase I study of adoptive transfer of these cells into patients with advanced, recurrent solid tumors besides malignant lymphoma (51). The results showed that the repetitive administration of *ex-vivo* expanded allogeneic NK cells was safe without any sign of graft vs. host disease or serious adverse event (51). Further studies are needed to enhance the persistence of these NK cells. Recently Jewett's group successfully expanded super-charged NK using PB-derived osteoclasts as feeder cells (52–54). These super-charged NK had superior cytotoxicity and IFN-γ secretion, survived for a longer period, and efficiently eliminated tumor growth in humanized xenografted mice (52–54). Considering more than 600,000 banked cord blood (CB) units worldwide (55), CB represents a unique opportunity as a readily available donor source with greater flexibility for the identification of HLA-compatible and KIR-mismatched lines. CB NK cells can be easily expanded with K562-mbIL21-41BBL feeder cells (18, 56) using CB mononuclear cells or they can be expanded to high log-scale with a cytokine cocktail from CD34+ CB progenitor cells (57, 58). NK cells derived from human CD34+ hematopoietic stem and progenitor cells showed efficient infiltration and killing of human ovarian cancer spheroids using an *in vivo*-like model system and reduced tumor progression in mice xenografted with ovarian carcinoma (59). NK cell lines also provide an unlimited source of effector cells and hold potential for development as standardized off-the-shelf therapeutics for adoptive cancer immunotherapy (60). Among different NK cell lines, NK-92 cells have been thoroughly investigated in preclinical studies and also been applied in clinical trials (61). The activated NK-92 based therapy from NanKwest was granted as the orphan drug designation against Merkel cell carcinoma. However, these NK-92 cell lines are aneuploid and must be irradiated before being administered to patients, which will limit the survival and proliferation of NK cells (62). In order to produce homogeneous and well-defined NK cells, a lot of effort has been put into generating NK cells derived from human embryonic stem cells or human induced pluripotent stem cells (iPSCs) (63–65). Most of these induced pluripotent stem cells-derived NK cells expressed no killer immunoglobulin (Ig)-like receptors (KIRs), which renders them unrestricted by recipients' HLA genotypes, and therefore they may serve as a universal “off-the-shelf” NK cell source for many recipients (64, 65).

## Stimulation of NK Cells Effector Function Using Cytokines and Cytokine Analogs

One of the earliest and most common approaches in using stimulating and activating NK cells for cancer immunotherapy has been the use of cytokines. Multiple cytokines and other novel soluble factors have been described in the literature to enhance the number, function and persistence of NK cells *in vivo*. IL-2, IL-15, IL-12, IL-18, and IL-21 have all been described in regulating NK cell function, particularly their activation, maturation and survival. Two most commonly employed strategies have been either pretreatment of NK cells with cytokines before the adoptive transfer, or administration of cytokines *in vivo*.

### IL-2

IL-2 is an immunostimulatory molecule first discovered in 1970s, and was initially described as a T cell growth factor (66). Further characterization showed the impaired NK cell function in IL-2 deficient mice, but NK cells were present in normal numbers in *IL-2* null mice suggesting that IL-2 is required for modulating NK cell function but is not essential for the development and maturation (67). To date, IL-2 has been the mostly commonly used cytokine in an attempt to boost NK cells *in vivo*. It was also the first every cytokine approved for clinical use (68). Earlier approaches using IL-2 in patient's involved using high dose IL-2 in conjunction with adoptive transfer of autologous NK cells. However, there was severe toxicity due to capillary leak syndrome related to IL-2 with no significant improvement in clinical outcomes (69). Subsequently, IL-2 was used to generate lymphokine activated killer cells, that were infused into the patients with melanoma or advanced RCC in combination with subcutaneous doses of IL-2 (70). This approach was not only well tolerated, and but clinical response showed a trend toward improved survival in melanoma patients who received a combination of lymphokine activated killer cells with IL-2 (5). In a phase I clinical trial involving patients with metastatic/unresectable digestive tract tumors, autologous NK cells were expanded *ex vivo* using IL-2, OK432, and modified recombinant human fibronectin fragment FN-CH296 induced T cells, and safely infused into the patients. However, no clinical responses were observed in these patients (71). Similarly, no improvement in clinical outcomes was observed in metastatic breast cancer patients who received IL-2 with autologous NK cell infusions following autologous SCT (72). Unfortunately, despite the compelling body of evidence suggesting that successful adoptive transfer and *in vivo* expansion of autologous NK cells when combined with IL-2 is safe and feasible, the clinical response against solid tumors has been minimal. It is likely that these impaired responses are related to the poor functional activation of the NK cells from cancer patients (73). Another potential explanation could be that IL-2 primed NK cells are sensitized to apoptosis upon coming in contact with the vascular endothelium likely causing a reduction in migration of these cells to the tumor site and infiltration into the tumor (74). Allogeneic or haploidentical NK cells infusions supplemented with IL-2 have been shown to produce significant clinical responses in hematological malignancies (75–77). However, such studies in field of solid tumors are lacking. In patients with advanced solid tumors, infusion of irradiated NK-92 cells that were *ex-vivo* expanded using IL-2 resulted in clinical responses in about three fourth of the patients with advanced lung cancer (78). Another phase II clinical trial using CD3 depleted, IL-2 stimulated haploidentical PBMC infusion in patients with recurrent ovarian and breast cancer showed a partial response in 20%, and stable disease in 60% of the patients (79). One of the major limitations of using IL-2 for modulating NK cell effector function, in addition to cytokine release with high dose IL-2 infusions, has been its ability to stimulate CD25 expressing Tregs (9). These Tregs have high affinity to IL-2 receptor, and diminish the effector response to NK cells by competing with NK cells for IL-2 and via TGFβ pathway (80). In a preclinical model, researchers have developed a mutant IL-2 molecule, “super-2,” that has increased affinity to IL-2Rβ and has been shown to have superior NK cell activation and proliferation compared to wild type IL-2. Additionally, it caused selective proliferation of cytotoxic T cells but not Tregs (81). Similarly, novel fusion protein molecules that combine NK cell activating receptor ligand with IL-2 are being developed to selectively promote the *in vivo* expansion and activation of NK without affecting Tregs (82). It is critical for future trials using IL-2 to adopt strategies that can circumvent these inhibitory elements like Tregs and myeloid suppressor cells to improve clinical responses.

### IL-15

Due to several potential drawbacks of IL-2 (as mentioned previously), IL-15 has emerged as an attractive alternative in cancer immunotherapy. IL-15 is a 15 kDa, gamma chain cytokine that possesses structural and functional similarities to IL-2, and is active in both cis- and trans- conformations (83). IL-15 receptor complex, which includes IL-15Rα/β/γ, wherein the β and γ chain receptor subunits are common to IL-2 and IL-15, and only difference exists at the α subunit. The relative high affinity between IL-15 and IL-15Rα, compared to IL-2 and IL-2Rα, results in NK cell activation at relatively lower doses. IL15Rα is expressed by a variety of immune cells like T cells, NK cells, natural killer T (NKT) cells, macrophages, and dendritic cells, and non-immune cells like skeletal muscle and endothelial cells (84, 85). *IL-15* deficient mice lack NK cells, NKT cells, memory CD8+ T highlighting that IL-15 is essential for development of these immune effector cells (86). In comparison to IL-2, IL-15 has a more potent effect on NK cell expansion, and it does not upregulated the gene expressions of type 2 cytokines like IL-6, IL-10, and IL-13 (87). Similarly when compared to IL-2, soluble IL-15 does not appear to expand Tregs (88). IL-15 has been shown improve functional abilities of NK cells by inducing granzyme and perforin through mTOR pathway, resulting in enhanced cytotoxicity (89–91). The antitumor effects of IL-15 have been well established in different preclinical studies (89, 92), and in part are mediated through the activating NK cells receptor NKG2D (93). IL-15 has been shown to enhance ADCC in a murine model of colon cancer, when given in combination with anti CD40 antibody (94). Higher levels of IL-15 on Day 15 post- autologous hematopoietic stem cell transplant (HSCT) have been shown to directly correlate with improved overall survival (OS) in patients with relapsed non-Hodgkin lymphoma (NHL) (95). Early patient studies with IL-15 were done in post HSCT setting or in patients with relapsed/refractory hematologic malignancies. A phase I dose escalation study in patients with acute myeloid leukemia using recombinant IL-15 with adoptively transferred NK cells showed that it was safe and feasible to administer IL-15, and it resulted in persistence and proliferation of NK cells *in vivo* (96). Patients with metastatic melanoma and metastatic RCC receiving *E coli* derived recombinant human IL-15 for 12 consecutive days showed 10-fold expansions of NK cells and a significant efflux of NK cells and memory CD8 T cells from peripheral blood further established that IL-15 infusions are safe and feasible (97). Another phase I study established the safety and clinical efficacy of allogeneic NK cell infusions cultured with IL-15 and hydrocortisone in patients with advanced non-small cell lung cancer (98). In a different phase I/II trial, four out of six patients with refractory pediatric solid tumor who received IL-15-stimulated NK cell infusion at 30 days after haploidentical-HSCT showed a clinical response (99). Other clinical trials using IL-15 alone or in combination with other immunotherapeutic agents targeting solid tumors are currently ongoing (NCT01572493, NCT03388632).

### IL-21

IL-21 is a type I cytokine synthesized by CD4+ T cells including NKT cells, T follicular helper and Th17 cells (100). It has been described to modulate both innate and adaptive immune responses, and is known to cause the lymphoid proliferation, particularly of CD8+ T cells and NK cells, and maturation of B cells (72). In addition to activating immune effector cells, IL-21 also plays a crucial role in mediating autoimmunity (101, 102). Binding of IL-21 to IL-21R primarily leads to activation of JAK1/JAK3 with subsequent phosphorylation of signal transducer and activator of transcription (STAT) (STAT3 and STAT1) signaling pathway resulting in upregulation of IFN-γ expression (103). However, IL-21 mediated activation can also occur via mitogen-activated protein kinase and phosphoinositide-3-kinase/serine/threonine kinase pathway. Combination of IL-21 and IL-15 has been shown to selectively promote the expansion of cytolytic CD56+CD16+ subtype of NK cells from human bone marrow (104). Using a K562 based antigen presenting cells genetically modified to express membrane bound IL-21 (mbIL-21), several thousand fold *ex vivo* expansion of NK cells can be achieved (49). Furthermore, these *ex vivo* expanded NK cells using mbIL-21 were found to have longer telomeres and higher expression of activating NK cell receptors. Multiple preclinical studies have established the powerful antitumor efficacy of IL-21 against solid tumors in mouse models. It has been shown to decrease tumor burden in mice bearing metastatic melanoma and RCC (105), melanoma and MethA fibrosarcoma (106), and head and neck squamous cell carcinoma (107). Several clinical trials have evaluated the safety, feasibility and antitumor effects of IL-21. Administration of recombinant IL-21 (rIL-21) has been shown to be safe with most common adverse event reporting grade1-2 toxicity, and severe toxicities requiring discontinuation being rare. A phase I study in patients with metastatic melanoma and RCC, rIL-21 at 30 μg/kg was well tolerated and shown to have antitumor activity, with about 70% patients showing some response or stable disease. One patient with melanoma achieved a complete remission (108), in a phase II study evaluating the efficacy and safety profile of IL-21 in patients, with metastatic melanoma, IL-21 was deemed safe and active against metastatic melanoma, with overall response rate being 22.5% and a favorable progression free and OS (109). Attempts to combine rIL-21 with targeted therapies have yielded mixed results. Combination of rIL-21 with sunitinib caused severe dose limiting toxicities with no clinical response resulting in early termination of the study (110). However, combining rIL-21 with sorafenib was shown to be relatively safe with mostly grade 1–2 toxicities, and was shown to have antitumor activity with objective response rate of 21% against metastatic RCC (111). Results are awaited from clinical trials evaluating the safety and efficacy of combining IL-21 with other immunotherapeutic agents (IL-21/Anti programmed cell death 1 [PD-1] against solid tumors/NCT01629758, IL-21/ipilimumab against melanoma, NCT01489059).

### IL-12

IL-12 is a heterodimeric, pro-inflammatory, type I cytokine that has been shown to elicit T-helper type-1 immune responses against infectious agents and cancer cells. It is mainly secreted by antigen presenting cells (macrophages and dendritic cells) and has been shown to promote the differentiation of CD4+Th^0^ cells into Th1 cells. It has been shown to increase cytokine production by NK cells and T cells, particularly IFN-γ (112). IL-12 does not appear to have any direct cytotoxic properties but exerts it's effects by stimulating NK and T cell proliferation and cytolytic properties (113), and by improving ADCC (114, 115). The antitumor efficacy of IL-12 has been well established in murine models in multiple preclinical studies (116–119). Despite the initial dose escalation phase I trial using recombinant human IL-12 (rhIL-12) establishing the safety of IL-12 administration in humans (120), subsequent phase II study had to be temporarily stopped due to severe toxicities, and deaths of the 2 patients (121). Subsequent studies have focused on establishing a safe dosing regimen for IL-12 administration to optimize the dose and frequency of IL-12 in patients. It was shown that a priming dose of IL-12 2 weeks prior can significantly decrease the toxicity of subsequent relatively high doses. Intratumoral injections of rhIL-12 have been attempted in patients with head and neck squamous cell carcinoma with activation of B cell compartment, and presence of tumor infiltrating B cells, that correlated with OS (122). Other delivery methods that have been tried are electroporation of plasmid DNA coding for IL-12 in patients with melanoma (123), and PEGylated IL-12 plasmid formulations in patients with gynecologic malignancies (124). To date, clinical benefits of IL-12 administration have been modest. However, significant clinical responses with IL-12 have been reported in patients with cutaneous T cell lymphoma (125) and in patients with acquired immune deficiency syndrome associated Kaposi sarcoma (126).

### IL-18

Similar to IL-12, IL-18 is another immunostimulatory cytokine belonging to IL-1 family that regulates both innate and adoptive immune responses. IL-18 is produced by monocytes, macrophages, neutrophils and dendritic cells, and is initially secreted in an inactive form pro-IL-18 which becomes biologically active upon cleavage by caspase-1 (127). IL-18 plays a key role in stimulating IFN-γ production from NK cells (128), and mice deficient in IL-18 have impaired cytotoxic responses, and decreases IFN-γ production (129). IL-18 has been shown to enhance TNF signaling in NK cells, prolonging the messenger ribonucleic acid (mRNA) expression of c-apoptosis inhibitor 2 and TNF receptor-associated factor 1 which inhibits NK cell death (130). *In vivo* antitumor efficacy of IL-18 has been well established in preclinical studies (131–133). However, there have only been few clinical studies evaluating its safety and efficacy in human subjects. Different phase I studies in patients with cancer have established the safety of rhIL-18 administration (134, 135). However, a subsequent study in patients with metastatic melanoma did not show any significant clinical responses as a monotherapy (136). Further studies evaluating its efficacy in combination with other cytokines and immunotherapeutic agents are required.

## Cytokine Analogs

### IL-15 Superagonist—ALT-803

Cytokine agonists have been well described in the literature, particularly for IL-15 (137). To further improve the biological activity and pharmacokinetics of a previously described IL-15 superagonist (IL-15N72D), investigators designed a novel molecule where IL-15N72D was fused with a dimeric IL-15 receptor a complex–(IL-15Rα/Fc). This redesigned IL-15 superagonist, ALT-803 has been shown to promote NK cell proliferation has been shown to possesses superior biological activity, higher potency and a much longer half-life (25 h vs. <40 min) compared to wild type IL-15 (138). Early preclinical studies showed that ALT-803 could upregulate the expression of NKG2D, promoted IFN-γ secretion and promoted the expansion of CD8^+^CD44^high^ memory T cells *in vivo* in a murine multiple myeloma model (139). Several other preclinical studies have established its efficacy in animal models against bladder cancer (140), B cell lymphomas (141), glioblastoma (142), breast, and colon cancer (143), and ovarian cancer (144). These antitumor effects have been attributed to increase in specific subpopulations of NK and memory CD8+ T cells, increased IFN-γ secretion and improvement in NK cell functionality. Early successes in preclinical studies have led to further investigation of ALT-803 in multiple clinical trials. A phase I trial in relapsed hematologic malignancies following SCT, ALT-803 induced clinical responses in 19% of the patients with one patient achieving complete remission. ALT-803 also induced proliferation and expansion of NK and CD8+ T cells in these patients (145). Another phase I trial in patients with advance solid tumors has established the safety and tolerability of ALT-803 administration (146). Combination of ALT-803 with nivolumab in patients with metastatic non-small cell lung cancer showed an objective response in 29% of the patients with 76% of the patients experiencing disease control. No dose limiting toxicities were seen in this trial (147). Several other clinical trials evaluating the antitumor effects of ALT-803 are currently ongoing (NCT03228667, NCT03127098, NCT03022825, NCT02384954, NCT02138734, NCT02890758, NCT02559674, NCT03520686).

### NKTR-255

NKTR**-**255 is another novel IL-15 analog that is currently undergoing preclinical development. NKTR-255 consists of a polymer-engineered IL-15 molecule that has been designed to optimally engage IL-15 receptor complex. In preclinical studies, it has been shown to have superior binding affinity to IL-15Ra and lower *in vivo* clearance (22 h vs. 1 h) in comparison to IL-15. It was also shown to induce phosphorylation of STAT5, decrease tumor burden in metastatic lung cancer mouse model and enhance the activation and proliferation of NK cells (148). The early results are exciting, and highlight its role as a promising immunotherapeutic agent. However, further studies are required at this time.

## Optimizing NK Cell Mediated ADCC

One of the principle ways NK cells exert their antitumor effects is through ADCC, where Fc portion of the antitumor antibody binds to FcγRIIIA and/or FcγRIIC expressed on NK cells, leading to the NK cell activation, and initiation of a series of events like transduction of death signals via TNF family death receptor signaling, release of cytotoxic granules from NK cells, and production of inflammatory cytokines like IFN-γ causing target cell killing (149). There are wide differences in the expression of activating and inhibitory receptors profile of NK cells amongst individuals. It is also well documented that polymorphisms between FcγRIIIA and FcγRIIC can influence the Fc receptor function. These polymorphisms result in a differential activation upon binding with an antitumor antibody. Patients with higher affinity polymorphisms have been shown to have superior outcomes with mAb treatment (150, 151). In order to augment the polymorphonuclear cell mediated ADCC, investigators have attempted to design an anti-human epidermal growth factor receptor-2 with tandem IgG1/IgA2 Fc that retains IgG1 FcγR binding but also provides the benefits of FcαRI/IgA Fc interactions. Their results showed that the tandem IgG1/IgA2 approach was superior in recruiting and engaging cytotoxic polymorphonuclear cells than either the parental IgG1 or IgA2 (152). Investigators have also attempted to improve the binding affinity of mAbs to maximize the ADCC. Obinutuzumab, a glycoengineered humanized anti-CD20 antibody has been shown to be superior to chimeric anti-CD20 mAb Rituximab in preclinical studies (153). By modifying the antibody backbone, it is possible to create chimeric antibodies (Ch14.18) with significantly longer half-life compared to the murine (mouse hybridoma 3F8), and avoid the human-mouse antibody response (154). An increasing number of humanized and fully human mAbs are currently being investigated in preclinical and clinical studies. Different combination strategies have been tried to improve antitumor ADCC of mAbs. NK cells have been shown to downregulate FcγRIIIA upon activation, and this downregulation is believed to be caused by activation of matrix metalloproteinases by the target cells (155). Preclinical studies have shown that ADAM17 inhibitor inhibits FcγRIIIA shedding and increased NK cell degranulation and IFNγ production (156). Strategies to increase the target antigen density on tumor cells for more efficient targeting by mAbs have been explored. Ionizing radiation (157) and Toll like receptor-9 agonists (158) have been shown to increase the expression of certain tumor target antigens. Currently there is limited preclinical data available about the clinical efficacy of these combinations and further studies are required.

## Preventing CD16 Shedding and Expressing High Affinity of CD16

CD16, also known as the human IgG Fc receptor III (FCγRIII), consists of two isoforms (CD16A and CD16B) (159). CD16A is a transmembrane protein and the only FcγR expressed by NK cells (159). It binds to IgG of an antibody and is essential for ADCC, which is a key mechanism of NK cells to lyse tumor cells (149). CD16B is mainly expressed on neutrophil cells (159). Both CD16A and CD16B are cleaved rapidly on neutrophil and NK cell activation after mitogen stimulation and co-culturing with tumor targets and the cleavage is mediated by a metalloprotease, ADAM17 (a disintegrin and metallopeptidase domain 17) (160, 161). The plasma levels of CD16 were significantly reduced in patients treated with an ADAM17 inhibitor (160, 161). The recent preclinical study demonstrated that the ADAM17 inhibitor BMS566394 significantly enhanced the expression of CD16 on NK cells and more importantly, it enhanced the cytotoxic activity and IFN-γ production of treated NK cells combined with trastuzumab against breast cancer cell lines (162). MEDI3622 is a human mAb of ADAM17 with high specificity and a potent inhibitory activity (163). The combination of MEDI3622 with anti-human epidermal growth factor receptor 2 (HER2) antibody trastuzumab greatly augmented the production of IFNγ by NK cells against ovarian cancer cell by blocking the shedding of CD16A on NK cells (164). Engineering NK cells with a CD16 mutant which has mutation(s) in the cleavage domain can also disrupt cleavage and prevent CD16 shedding. Expression high affinity CD16 FcγRIIIa in NK cells is another attractive choice. The insertion of the high affinity CD16 FcγRIIIa (158V) allele and IL-2 into NK-92 cells render NK-mediated ADCC using cetuximab, trastuzumab and pertuzumab against a variety of solid tumor cells (165). Additional strategies include engineering NK cells with chimeric receptors CD16-BB-ζ and CD64-BB-ζ (166). These engineered NK cells significantly improved cytotoxicity against CD20-positive NHL cells in the presence of rituximab (166) but their anti-tumor effects need to be evaluated for solid tumor cells with targeted antibodies.

## Role of Immunocytokines in Improving NK Cell Mediated Cytotoxicity

As previously described in this review, a variety of cytokines have been utilized in an attempt to improve NK cell function and stimulation. Early clinical trials have demonstrated the improvement in outcomes in pediatric patients with neuroblastoma that received immunotherapy with anti-GD2 ch14.18 antibody in combination with IL-2 and granulocyte-macrophage colony-stimulating factor (167), whereas no clear benefit of antibody treatment without cytokine support was observed in a similar study performed by a German group suggesting a beneficial role for combining antibody therapy with cytokines (168). However, this approach has had mixed responses with limited clinical success against solid tumors. This is partly due to the challenges with systemic administration of these cytokines. Systemic cytokines have a narrow therapeutic window limiting their efficacy and they can cause severe toxicities by increasing the vascular permeability from a cytokine storm. These limitations have fueled the development of immunocytokines that are novel fusion proteins created by linking tumor specific mAbs to cytokines. The antibody component directs the cytokine molecule to the tumor location with selective activation of cytokine molecules at the site of antitumor activity. Studies have shown that treatment with immunocytokines leads to the targeted increase in the density of NK cells and lymphocytes in the tumor extracellular matrix (169, 170). Several immunocytokines molecules have shown promise in preclinical studies. Anti-GD2-IL2 fusion immunocytokine has been shown to have superior antitumor efficacy against neuroblastoma compared to both molecules administered separately at the same time. The mechanism was reported to be exclusively NK cell mediated (171). Similarly, anti-GD2-RLI (an IL-15 superagonist) fusion showed improved half-life of RLI and was effective against metastatic NXS2 neuroblastoma in a syngeneic mouse model (172). A fusion protein between tumor necrosis-targeting human IgG1 NHS76 and IL-12 (NHS-IL12) had longer half-life *in vivo*, stimulated lower IFN-γ release by immune cells thereby limiting the IL-12 mediated toxicity, and had superior antitumor efficacy in mouse models (173). Further modifications of IL-2 based immunocytokines have been attempted, e.g., single IL-2 variant (IL2v) moiety with loss of CD25 binding, to avoid Treg stimulation and improve the targeted biological activity (174). Several of these molecules have been tested in clinical trials. In a phase II clinical trial of hu14.18-IL2, complete resolution of bone marrow disease and metaiodobenzylguanidine avid disease was seen in 5 out of 24 Stratum-2 patients with relapsed-refractory neuroblastoma (175). Phase I/II clinical trials have established the safety of intravenous administration of TNF-IL2 fusion protein (L19-TNF) in patients with advanced solid tumors (176), and it was shown to have clinical efficacy in patients with advanced localized melanoma in combination with melphalan and mild hyperthermia (177). More recently, phase I trial of NHL-IL12 established safety in patients with metastatic solid tumors. Evaluation of peripheral immune cell subset showed an increase in activated and mature NK and NKT cells in these patients (178). These agents have shown a great promise in stimulating immune cells like NK cells and cytotoxic T cells locally at the tumor site with cytokine component while maintaining the targeted effector antibody response. Multiple ongoing clinical trials are evaluating the safety and efficacy of several other immunocytokines alone, and in combination with other therapeutic modalities like immune checkpoint inhibitors (NCT03209869, NCT03386721, NCT02627274, NCT02350673). Table 2 provides a comprehensive list of past and current clinical trials evaluating the safety and efficacy of immunocytokines against solid tumors.

**Table 2:** Clinical development of immunocytokines/fusion proteins against solid tumors.

**Immunocytokine**	**Malignancy**	**Combination**	**Phase**	**Registry**
Hu14.18-IL2	Neuroblastoma/GD2+ Tumors	Monotherapy	I	NCT00003750
Hu14.18-IL2	Relapsed/refractory neuroblastoma	*Ex vivo* expanded NK Cells	I	NCT03209869
Hu14.18-IL2	Recurrent neuroblastoma	Sargramostim and Isotretinoin	II	NCT01334515
Hu14.18-IL2	Melanoma	Monotherapy	II	NCT00109863
Hu14.18-IL2	Residual/refractory neuroblastoma	Monotherapy	II	NCT00082758
L19-TNFα	Stage III/IV limb melanoma	Melphalan	I	NCT01213732
L19-TNFα	Advanced solid tumors	Doxorubicin	I	NCT02076620
L19-TNFα	Solid tumors, colorectal cancer	Monotherapy	I/II	NCT01253837
L19-TNFα	Unresectable or metastatic soft tissue sarcoma	Doxorubicin	I	NCT03420014
F16-IL2	Solid tumors	Doxorubicin	I/II	NCT01131364
F16-IL2	Solid tumor, breast cancer, melanoma, NSCLC	Paclitaxel	I/II	NCT01134250
L19-IL2	Advanced solid tumors, RCC	Monotherapy	I/II	NCT01058538
L19-IL2	Oligometastatic solid tumor	SABR	I	NCT02086721
L19-IL2	Metastatic melanoma	Dacarbazine	I/II	NCT02076646
L19-IL2	Advanced/metastatic pancreatic cancer	Gemcitabine	I	NCT01198522
NHS-IL2LT	Metastatic or locally advanced solid tumors	Cyclophosphamide	I	NCT01032681
NHS-IL2LT	NSCLC	Local Tumor Irradiation	I	NCT00879866
NHS-IL12	Epithelial/malignant mesenchymal tumors	Monotherapy	I	NCT01417546
NHS-IL12	Advanced solid tumors	Avelumab	I	NCT02994953
CEA-IL2v (RO6895882)	Advanced/metastatic solid tumors	Monotherapy	I	NCT02004106
CEA-IL2v (RO6895882)	Locally advanced/metastatic solid tumors	Atezolizumab	I	NCT02350673
FAP-IL2v (RO6874281)	Solid tumors, breast cancer, head and neck cancer	Trastuzumab Cetuximab	I	NCT02627274
FAP-IL2v (RO6874281)	Advanced and metastatic solid tumors.	Atezolizumab Gemcitabine Vinorelbine	II	NCT03386721
huKS-IL-2 (EMD273066)	Refractory epithelial carcinoma	Monotherapy	I	NCT00016237
huKS-IL-2 (EMD273066)	Ovarian, prostate, colorectal, NSCLC	Cyclophosphamide	I	NCT00132522
BC1-IL12 (AS1409)	Metastatic malignant melanoma, RCC	Monotherapy	I	NCT00625768
LAG3-Ig (IMP321)	NSCLC head and neck cancer	Pembrolizumab	II	NCT03625323
VEGF-Ig (HB002.1T)	Solid tumors	Monotherapy	I	NCT03636750
PAP-GM-CSF *(*APC8015)	Prostate cancer	Bevacizumab	II	NCT00027599

## ANIT-KIR Antibodies for Improvng NK Cell Cytotoxicity

As mentioned previously, NK cells remain tolerant to cells expressing HLA class I ligands but trigger cytotoxicity against altered cells that have a decreased level of HLA expression. This distinction between self and altered cells is mediated through inhibitory KIRs on NK cell surface. KIRs can recognize HLA molecules triggering inhibitory signals and resulting in decreased ADCC by NK cells. NK cells herald the immune recovery of lymphocyte subsets following allogeneic HSCT, and have been implicated in early graft vs. malignancy effects (35). This concept has been exploited clinically in allogeneic HSCTs for hematologic malignancies where donor KIR is mismatched with recipient's tumor creating a KIR-ligand incompatibility in order to create graft vs. leukemia effect (179). Similar to the KIR-ligand mismatch concept, investigators have designed mAbs that block the HLA-KIR interactions to prevent the NK cell inhibition and trigger cytotoxicity. Phase I clinical trial with IPH2101, the first in class anti-KIR antibody that inhibits KIR2DL-1, L-2, and L-3, in patients with relapsed/refractory multiple myeloma established the safety at dose that achieve full inhibitory KIR saturation (180). In another phase I study for relapsed multiple myeloma, a combination of IPH2101 with lenalidomide resulted in objective responses in five out of 15 patients, with median progression free survival being 24 months (181). In preclinical studies, the second generation fully human IgG4 anti-KIR2DL1, -L2, -L3, -S1, -S2 antibody (IPH2102/Lirilumab) was shown to potentiate the spontaneous cytotoxicity of NK cells against lymphoma cells lines. It was also shown to augment the NK cells mediated ADCC with Rituximab against CD20 lymphoma cells, *in vitro* and *in vivo* (182). Very few studies have looked at the efficacy of these anti-KIR antibodies against solid tumors. IPH2102 was well tolerated in patients with solid tumors and hematologic malignancies, with patients experiencing only mild and transient side effects (183). A recently published study established a correlation between the expression of inhibitory KIR and PD-1 on tumor cells in patients with non-small cell lung cancer suggesting a potential benefit of combining anti-KIR antibodies with anti-PD-1 treatment to circumvent the immune escape in these patients (184). Several active studies are currently evaluating these anti-KIR antibodies against solid tumors in combination with other immune therapies (NCT03341936, NCT03203876, NCT03347123).

## Re-Directing NK Cells With Chimeric Antigen Receptor (CAR)

**Figure 3:**
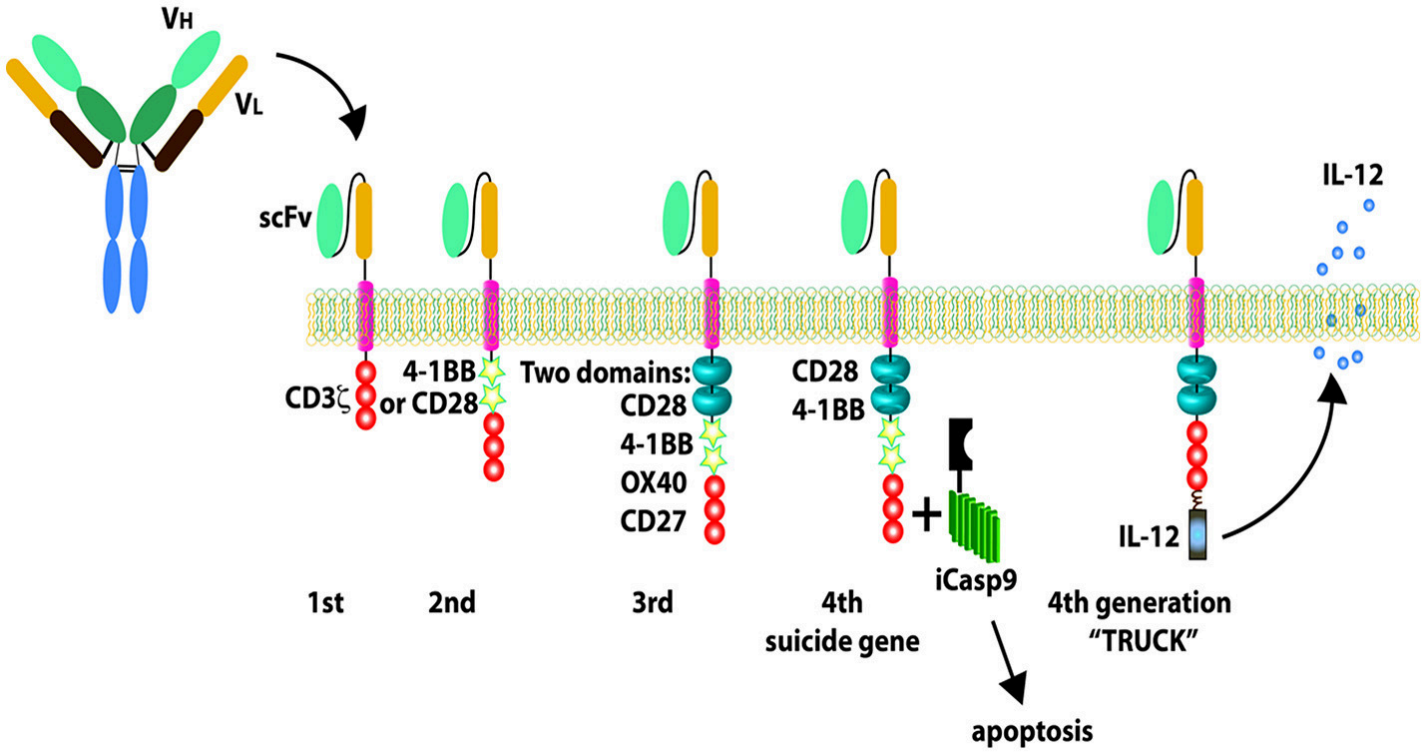
Chimeric antigen receptors (CARs). The first-generation CARs only have CD3 zeta signaling domain; the second-generation CARs include one CD28 or 4-1BB co-stimulatory components combined with CD3 zeta signaling domain; the third-generation CARs include two co-stimulatory domains; the fourth-generation CARs are designed with new elements including a controllable suicide gene like caspase 9 or loaded with IL-12 secretion. Reproduced with permission from: Barth et al. (39).

The adoptive transfer of T cells engineered to express an artificial CAR to target a specific antigen on tumor cell surface is an exciting approach for cancer immunotherapy. CARs usually include a single-chain variable fragment from a mAb, a transmembrane hinge region, and a signaling co-stimulatory domain such as CD28, CD3-zeta, 4-1BB (CD137), or 2B4 (CD244) endodimers (185–187). The co-stimulatory components attribute greater strength of signaling, and longer *in vivo* T-cell persistence (39). Four generations of CAR have been developed and evaluated pre-clinically and clinically (39) (Figure 3). The advantage of the CAR strategy is that no HLA expression on the target cell is required for the epitope to be accessible to CAR^+^ immune cells. Thus, CAR^+^ immune cell application is not limited to only a subset of patients with a specific HLA type (185–187). To increase the targeting specificity of expanded NK cells, our group has investigated functional activities of peripheral blood natural killer cells modified by mRNA nucleofection with anti-CD20 CAR against CD20+ B-NHL *in vitro* and in xenografted NSG mice (188). Lentiviral transduced methods had been used to generate CAR expressing NK cell lines targeting solid tumor cells. The CARs have been developed and engineered in NK cells lines against several antigens for solid tumors which include epidermal growth factor receptor (EGFR), HER2, EGFRvIII, GD2, epithelial cell adhesion molecule (EpCAM) (Table 3) with efficiency in preclinical studies. Schönfeld et al. generated a stable NK92 cell line expressing a humanized anti-HER2 CAR containing CD28 and CD3ζ signaling domains and these CAR NK cells efficiently lysed HER2+ tumor cells *in vitro* and the specific recognition of tumor cells resulted in selective enrichment of anti-HER2 CAR NK-92 cells in orthotopic breast carcinoma xenografts and reduction of pulmonary metastasis in a RCC model, respectively (189). In another study, the repeated stereotactic injection of anti-HER2 CAR NK-92 cells improved the symptom-free survival in glioblastoma xenografted mice (190). NK-92 cells and primary NK cells were engineered to express the second generation of EGFR-CAR to target breast cancer cells (191). *In vitro*, compared with mock-transduced NK-92 cells or primary NK cells, EGFR-CAR-engineered NK-92 cells and primary NK cells displayed enhanced cytotoxicity and IFN-γ production when co-cultured with breast cancer cell lines (191). In the mice intracranially pre-inoculated with EGFR-expressing breast cancer cells, intratumoral administration of EGFR-CAR-transduced NK-92 cells mitigated tumor growth compared to mock NK cells (191). A human NK cell line KHYG-1 expressing anti- EGFRvIII CAR was established and exhibited the inhibition of glioblastoma cell-growth via apoptosis in an EGFRvIII-expression specific manner (192). Another group engineered NK-92 to stably express an anti-GD2 CAR and these CAR NK-92 cells facilitated tumor effective recognition and elimination of GD2^+^ NB cell lines and primary NB cells (193). Anti-EpCAM CAR engineered NK-92 displayed high and selective cell-killing activity against EpCAM-expressing breast carcinoma cells that were resistant to the natural cytotoxicity of unmodified NK cells (194). Additionally, our group is developing anti-ROR1 CAR engineered expanded primary NK cells through CAR mRNA electroporation technology to target ROR1^+^ solid tumors with promising *in vitro* anti-tumor effects (195). Anti-mesothelin CAR-NK cells were derived from CAR-expressing iPSCs with the optimized CAR construct (197). These CAR-NK cells showed great potent ability to kill mesothelin-expressing tumors both *in vitro* and *in vivo*, demonstrating a potential strategy to produce “off the shelf,” targeted allogeneic cell products for refractory malignancies (197). Besides designing a CAR based on the single chain variable fragment (scFv) of a mAb again an antigen on tumor cell surface, CAR can also be formed from a NK activating receptor such as NKG2D followed by transmembrane domain and signal transduction domains. Chang et al. designed a CAR termed NKG2D-DAP10-CD3ζ that was composed of the NK cell activating molecule NKG2D plus 2 key signaling molecules, DAP10 and CD3ζ (196). These NKG2D CAR engineered primary NK cells through retroviral transduction showed significantly enhanced *in vitro* cytotoxicity against a variety of solid tumor cell lines that express NKG2D ligands MICA/B such as the osteosarcoma cell lines U-2 OS, MG-36, HOS, the prostate carcinoma cell lines DU 145 and PC-3, and the rhabdomyosarcoma cell line RH36 (196) and significantly reduced tumor burden in osteosarcoma xenografted NSG mice compared to mock NK cells (196). Similar strategy can be applied to generate other NK activating receptor based CAR like NKp30-CAR to enhance NK cytotoxicity. The advantage of this CAR strategy is that one CAR can be applied for a variety of tumor types in a matter expressing the corresponding ligands. Considering the recent safety concerns such as cytokine release syndrome and neurotoxicity associated with infusion of CAR-modified T cells (187), a suicide gene should be incorporated into the construct as a safety measure for CAR NK therapy but it is debatable because of the short life span of NK cells compared to T cells. Additionally, IL-15 secretion CAR-NK can be generated retroviral transduction by incorporating IL-15 to CAR design (18) to enhance the CAR NK proliferation, persistence and homing in solid tumors.

**Table 3:** Summary of CAR NK in preclinical studies for solid tumors.

**Antigen**	**NK resource**	**CAR signaling domains**	**Methods**	**Diseases**	**Report year**	**References**
ErbB2/HER2	NK92	CD28 and CD3ζ	Lentiviral transduction	Breast carcinoma	2015	(189)
ErbB2/HER2	NK92	CD28 and CD3ζ	Lentiviral transduction	Glioblastoma	2015	(190)
EGFR	NK92 and primary NK	CD28 and CD3ζ	Lentiviral transduction	Breast cancer brain metastases	2016	(191)
EGFRvIII	KHYG-1	N/A	Lentiviral transduction	Glioblastoma	2018	(192)
GD2	NK92	CD3ζ	Retroviral transduction	Neuroblastoma	2012	(193)
EpCAM	NK92	CD28 and CD3ζ	Lentiviral transduction	Breast carcinoma	2012	(194)
ROR1	*Ex vivo* expanded human NK	4-1BB and CD3ζ	CAR mRNA electroporation	Neuroblastoma, sarcomas	2017	(195)
NKG2D	*Ex vivo* expanded human NK	DAP10 and CD3ζ	Retroviral transduction	Osteosarcoma, prostate carcinoma, rhabdomyosarcoma	2013	(196)
Mesothelin	iPSCs-NK	NKG2D-2B4-CD3ζ, NKG2D-2B4-DAP10-CD3ζ, NKG2D-4-1BB-2B4-CD3ζ	Piggybac transposon integration	Mesothelin+ solid tumors such as ovarian cancer	2018	(197)

There are currently 3 registered clinical trials testing the safety and efficacy of CAR-NK cells in patients with solid tumors. One trial is a single-center, single arm, open-label pilot study to evaluate the safety and feasibility of CAR-NK cell treatment in subjects with metastatic solid tumors using autologous or allogeneic NK cells transfected by mRNA electroporation against NKG2D-ligand expressing cancer cells (NCT03415100). Another trial is to evaluate the efficacy and safety of CAR-modified NK Cell lines in MUC1 positive advanced refractory or relapsed solid tumors (NCT02839954). These two trials are being conducted in China. The third trial is being conducted in the USA sponsored by Johann Wolfgang Goethe University Hospital to evaluate the safety and tolerability of NK-92/5.28.z (HER2.taNK) for patients with recurrent HER2-positive glioblastoma (NCT03383978). Pharmacokinetics and pharmacodynamics and potential signs of anti-tumor activity of NK-92/5.28.z cells will also be analyzed.

## Bispecific Antibodies to Enhance NK Cell Killing Potential

mAbs have revolutionized the development of anticancer therapeutics over past last few decades. However, the efficacy of mAbs has been limited against solid tumors. Advances in protein engineering has made the generation of bispecific molecules possible. Bispecific antibodies are novel molecules where two antigens can be targeted at the same time by combining the specificities of two antibodies. The design of a bispecific antibody constitutes an antitumor scFv targeting a specific malignancy is linked to an anti-CD3/anti-CD16 in order to create an immune connection between cancer cell and the immune effector cells like T cell or NK cell. Recently, there has been a growing interest in development of bispecific antibodies with currently multiple studies evaluating their anti-cancer potential in preclinical and clinical studies. To date, the most success with bispecific antibodies has been seen with T cell specific bispecific molecules like catumaxomab (CD3/EpCAM) against malignant ascites, and blinatumomab (CD3/CD19) and ionotuzumab (CD3/CD22) against B cell lymphoblastic leukemia (198–200). These successes have encouraged the development of diverse bispecific antibodies with varied clinical applications besides cancer, like emicizumab/ACE910 for patients with Hemophilia A. The goal of developing these bispecific engagers is to enhance the therapeutic efficacy, improve targeted delivery to the tumor site, optimize immune cell engagement, and reduce off target effects and relative ease of administering one drug instead of two separate molecules. One major shortcoming of the T cell specific antibodies has been their potential to cause massive cytokine release causing capillary leak, hypotension and respiratory distress in a clinical setting. These shortcomings have made NK cell based bispecific NK cell engagers an attractive alternative. Bispecific NK cell based antibodies can engage the Fc portion of the antibodies through their FcγRIII (CD 16) receptor with the other portion designed to bind a specific epitope on the tumor surface. Several NK based bispecific antibodies are currently in preclinical and clinical development. Early investigations have focused on AFM13, an anti-CD30/CD16A for relapsed or refractory Hodgkin lymphoma. In Phase I trial, AFM13 was shown to be safe, caused activation of NK cells, decreased soluble CD30 in peripheral blood, was found to be active in patients resistant to brentuximab, and achieved disease control in 77% patients at doses ≥1.5 mg/kg (201). Phase II studies with AFM13 are currently ongoing. Multiple preclinical studies are evaluating different bispecific and trispecific NK cell engaging antibodies against solid tumors. A trivalent bispecific antibody targeting ErbB2 and CD16 was shown to be more potent than anti-ErbB2 single-chain variable fragment (scFv)-Fc fusion protein *in vitro* against breast cancer cell lines, and *in vivo* against breast cancer xenograft mouse model (202). Multiple other antibodies targeting the HER2- FcγRIII antigens have been described (203–205). Similarly, a completely humanized bispecific antibody targeting EpCAM and CD16 showed significant increase in ADCC, increased degranulation of NK cells with concomitant increase in IFN-g production against EpCAM positive prostate, breast, colon, and head and neck cancer cell lines (206). Modifications have been made to the dimeric structure of these bispecific antibodies to further improve the efficacy. A tribody targeting human epidermal growth factor 2 where two HER2-specific scFvs were linked to CD16 [(HER2)_2_xCD16] was found to be superior to trazutumab against HER2-expressing breast, pancreatic, ovarian, and esophageal tumor cells with increased NK cell degranulation and release of granzyme B (207). Insertion of a modified interleukin-15 cross-linker to an EpCAM/CD16 bispecific construct to create a trispecific construct improved NK cell proliferation and survival and showed increased ADCC (208). Table 4 highlights multiple CD16 targeting bispecific and trispecific antibodies that have undergone preclinical development. The results of these preclinical studies are encouraging and warrant further clinical development of these molecules.

**Table 4:** Preclinical development of CD16 antigen based bispecific antibodies targeting solid tumors.

**Bispecific antibody**	**Target malignancy**	**References**
Anti-MOV19-AntiCD16	Ovarian cancer	Ferrini et al. (209)
Anti-ovarian carcinoma(IgG2a)-AntiCD16	Ovarian cancer	Ferrini et al. (210)
Anti-NCAM -AntiCD16	Human glioma	Obukhov et al. (211)
Anti-CA19-9- AntiCD16	Colorectal adenocarcinoma	Garcia de Palazzo et al. (212)
Anti-c-erbB-2-Anti CD16	Ovarian cancer	Weiner et al. (213)
Anti-FBP-Anti CD16	Ovarian cancer	Ferrini et al. (214)
Anti-c-erbB-2-Anti FcgammaRIII	c-ERB-2 positive tumors	Weiner et al. (215)
Anti-c-erbB-2-Anti FcgammaRIII	Ovarian adenocarcinoma	Weiner et al. (216)
Anti-HER2/neu- AntiFcgammaRIII	Ovarian adenocarcinoma	McCall et al. (217)
Anti-PDGFR- AntiFcgammaRIII	Lung cancer	Gruel et al. (218)
Trivalent anti-erbB2-anti-CD16	Breast cancer	Xie et al. (219)
Anti-HER2/neu-Anti CD16 minibody	Ovarian cancer	Shahied et al. (220)
Anti-ErbB2-Anti-CD16	Breast cancer	Lu et al. (202)
Anti-EGFR-Anti-CD16	Cholangiocarcinoma	Asano et al. (221)
Anti-EpCAM-Anti-CD16	Prostate, breast, colon, head and neck cancer	Vallera et al. (206)
Anti-HER2-Anti- FcγRIIIA	Breast cancer	Turini et al. (205)
Anti-CD133-Anti-CD16	Colorectal cancer	Schmohl et al. (222)
Anti-CEA and Anti-CD16	Ovarian and colorectal cancer	Dong et al. (223)
Anti-EpCAM-IL-15 crosslinker-Anti-CD16 (TriKE)	Colorectal cancer	Schmohl et al. (208)
Anti-EpCAM-AntiCD133-IL-15 crosslinker-Anti-CD16 (TetraKE)	Colorectal cancer	Schmohl et al. (224)
AntiCD133-IL-15 crosslinker-Anti-CD16 (TriKE)	Colorectal cancer	Schmohl et al. (225)
Anti-Muc1-Anti-CD16	Colorectal, ovarian and lung cancer	Li et al. (226)
Anti-(HER2)_2_-Anti-CD16 Tribody	Pancreatic, ovarian, esophageal cancer	Oberg et al. (207)
Anti-GPC3-Anti-CD16	Liver carcinoma	Wang et al. (227)

## Targeting NK Cell Checkpoints PD-1, TIGIT, and IL-1R8

Immune checkpoints are negative regulators of immune cells, especially T cells, to help keep immune responses in check, and maintain self-tolerance during immune responses (228). Malignant cells often express high level of ligands of checkpoint inhibitory receptors, and escape from immune recognition and elimination (228). In recent years, the application of mAbs directed against immune checkpoint receptors or ligands has greatly enhanced the anti-tumor activity of the immune cells, and has resulted in remarkable clinical benefits (229, 230). Similar to T cells, NK cells also express an array of immune checkpoints which include PD-1, cytotoxic T-lymphocyte-associated protein 4, T cell immunoglobulin- and mucin-domain-containing molecule 3, T cell immunoreceptor with Ig and immunoreceptor tyrosine-based inhibition motif (ITIM) domains (TIGIT), CD96, lymphocyte activation gene-3, and IL-1R8 besides the well-known NK inhibitor receptors: KIRs and CD94/NKG2A (231–233). The data of the cytotoxic T-lymphocyte-associated protein 4, lymphocyte activation gene 3 and mucin-domain-containing molecule 3 on NK cells functions are either scarce or controversial. But several lines of evidences strongly demonstrate the inhibitory roles of PD-1, TIGIT, and IL-1R8 on NK cells. PD-1^+^ NK cells are confined to CD56dimNKG2A–KIR+CD57+ mature NK population and are functionally exhausted, exhibiting reduced proliferative capability, poor cytolytic activity and impaired cytokine production as compared with the PD-1^−^ NK cells (234, 235). A recent study demonstrated that the increased PD-1 expression on peripheral and tumor infiltrating NK cells from patients with digestive cancers indicates poorer prognosis (236). And blocking PD-1/PD-L1 signaling markedly enhances cytokines production and degranulation and suppresses apoptosis of NK cells *in vitro* (236). More importantly, a PD-1 blocking antibody was found to significantly suppress the growth of xenografts in nude mice, and this inhibition of tumor growth was completely abrogated by NK depletion, strongly suggesting that PD-1 is an inhibitory regulator of NK cells in digestive cancers (236). PD-1 blockade might be an efficient strategy in NK cell-based tumor immunotherapy. A phase II clinical trial is on-going to assess the effect of pembrozilumab (a humanized anti-PD-1 mAb) on NK cell function and exhaustion in melanoma (NCT03241927). TIGIT competes with the NK activating receptor DNAX Accessory Molecule-1 (CD226) for their common ligands CD112 (PVRL2) and CD155 (PVR) to directly dampen NK cell cytotoxicity (237). *In vitro* TIGIT blockade improves the anti-tumor effect of Trastuzumab (a recombinant humanized anti-HER2 mAb), which partially relies on NK cell-mediated ADCC (238). Recent evidence showed the upregulation of TIGIT on tumor-infiltrating NK cells in mouse models of subcutaneously administered solid tumors and the TIGIT expression on tumor-infiltrating NK cells was associated with tumor progression and was linked to functional exhaustion of NK cells (233). The blockade of TIGIT via mAbs reversed the exhaustion of anti-tumor NK cells in multiple tumor models, enhanced the infiltration of activated (CD69+) NK cells into tumors and thereafter improved the OS of the host (233). The presence of NK cells was critical for the therapeutic effects of blockade of TIGIT or the PD-1 ligand PD-L1 or combined blockade of both checkpoints (233). These findings demonstrate that the NK cell–associated TIGIT signaling pathway has a role in tumors' evasion of the immune system and that reversing NK cell exhaustion is critical for the therapeutic effects of anti-tumor immunotherapy based on the blockade of TIGIT (239). IL-1R8, also known as toll-interleukin 1 receptor or Single Ig IL-1-related receptor, is a member of interleukin-1 receptor family (IL1Rs) and acts as a negative regulator of IL1Rs and toll-like receptors (TLRs) to suppress ILR- and TLR-mediated cell activation (240). IL-1R8 is widely expressed in several epithelial tissues, in particular by epithelial cells of the kidney, digestive tract, liver, lung, lymphoid organs, and it is also expressed on monocytes, B and T lymphocytes, dendritic cells, and NK cells (240). Recently, Molgora et al. identified IL1R8 as a checkpoint protein in NK cells that regulates antitumor activity of NK cells in solid tumors (232). Utilizing IL-1R8-deficient (Il1r8–/–) mice as a study model, Molgora et al. found that IL1R8- deficient NK cells expressed significantly higher levels of the activating receptors NKG2D, DNAX Accessory Molecule-1 and Ly49H and fas ligand and produced increased levels of IFNγ and granzyme B (232). IL-1R8 partial silencing in human peripheral blood NK cells with small interfering RNA was associated with a significant increase in IFNγ production and upregulation of CD69 expression (232). In a model of sarcoma (MN/MCA1) spontaneous lung metastasis, *Il1r8*–/– mice showed a reduced number of hematogenous metastases, whereas primary tumor growth was unaffected and the protection was completely abolished in NK-cell-depleted *Il1r8*–/– mice (232). Additionally, adoptive transfer of *Il1r8*–/– NK cells significantly and markedly reduced the number and volume of lung and liver metastases in in the mice with MC38 colon carcinoma liver metastasis while *Il1r8*+/+ NK cells had no effect (232). These results suggest IL-1R8 serves as a negative regulator of NK cells and its inactivation unleashes human NK-cell effector function (232).

## Focusing on NKG2D and the Ligands

**Figure 4:**
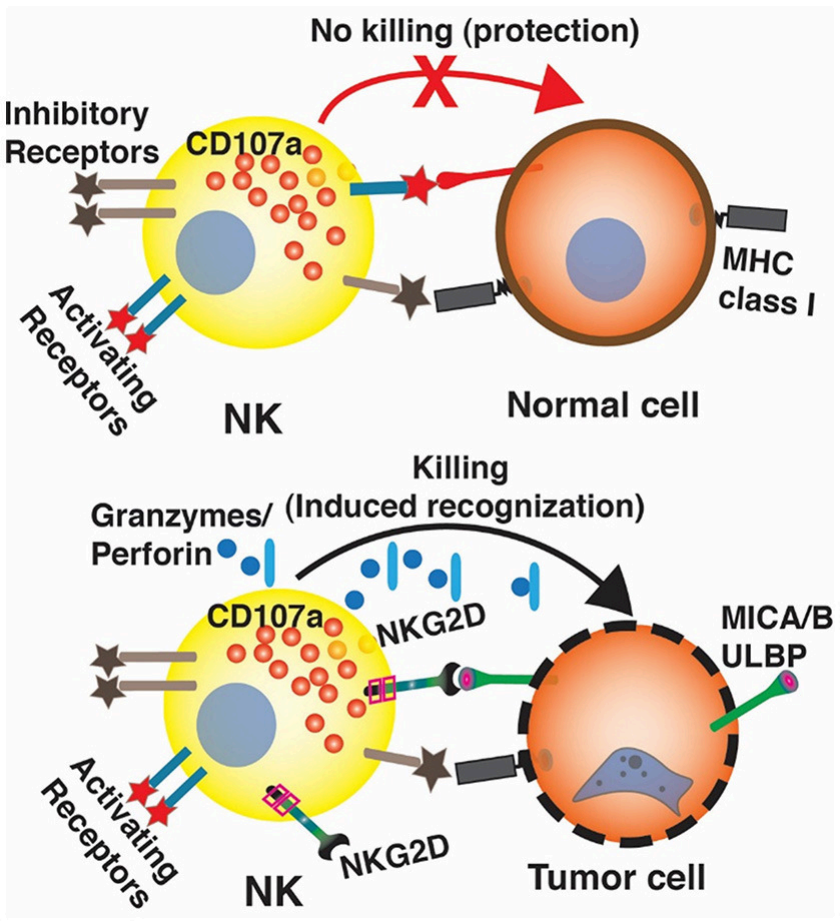
The interaction between NKG2D on NK cells and NKG2D ligands on tumor cells. In normal cells, NKG2D ligands express is very low. The functions of NK cells are balanced by the signals from the inhibitory and activating receptors. In humans, when normal cells are transformed into cancer cells. NKG2D ligands such as MICA/B and ULBP proteins, are often overexpressed. The engagement of NKG2D and NKG2D ligands overcomes inhibitory signals on NK cells, activates NK cells to release cytotoxic molecules such as perforin and granzyme, and trigger apoptosis of tumor cells.

NKG2D is a C-type, lectin-like, type II transmembrane glycoprotein-activating receptor expressed in humans on NK, natural killer T, activated CD8+ T cells and some CD4+ and γδ+Tcell subsets (241). In humans, NKG2D forms a hexameric structure with the adaptor molecule DNAX-activating protein of 10 kDa (DAP10) to mediate signal transduction and cellular activation upon ligand recognition (242). NKG2D ligands are structural homologs of MHC class I molecules and are upregulated on the surface of many cell types by cellular stress and viral/bacterial infections and frequently during tumorigenesis (243, 244). The currently identified human NKG2D ligands include MICA and MICB and UL16 binding protein (ULBP1–ULBP6) families (245). Several lines of evidence conclusively demonstrated that engagement of NKG2D and NKG2D ligands, such as MIC A/B elicits cytolytic responses overcoming inhibitory signals on NK cells and is sufficient to trigger cytolysis by NK cells expressing NKG2D (246–249) (Figure 4). The expression of these ligands on the tumor cell surface are regulated at multiple levels: transcriptional regulation, ribonucleic acid (RNA) splicing, posttranscriptional regulation, posttranslational regulation (245). NKG2D ligands can be cleaved from the tumor cell surface after translation by membrane matrix metalloprotease and be released as soluble ligands (245). The findings from “humanized” transgenic animal studies demonstrated the opposite roles of membrane-bound and soluble forms of NKG2D ligands (250, 251). The membrane-bound ligands binding to NKG2D play an important role in NK cell activation and tumor immune surveillance (247, 252, 253), while the soluble NKG2D ligands suppress tumor immunity by passively blocking NKG2D and inducing receptor internalization to down-regulate NKG2D on the surface of NK cells (254–256). Serum levels of soluble NKG2D ligands significantly correlate with patients prognosis and are used as prognostic markers in some tumor patients (257, 258). Therefore, therapeutic strategies have focused on enhancing NKG2D expression and signaling on NK cells such as expression of NKG2D CAR and applying IL-15 agonist as we discussed in the earlier section; enhancing the level of membrane-bound NKG2D ligand on tumor cells; and eliminating soluble NKG2D ligands (Table 5). We and others had utilized histone deacetylase inhibitors such as romidespin, entinostat, sodium valproate to enhance NKG2D ligands expression on tumor cell surface to enhance NK based immunotherapy (259, 260, 263, 264). Zhu et al. found that entinostat not only increased the expression of MICA/B on osteosarcoma cells but also simultaneously increased the expression of NKG2D on primary human NK cells to augment the activation pathways for NK cell recognition of cancer cells (259). Their results indicate that entinostat has the potential to enhance concurrent NK-cell therapy for solid tumors such as colon carcinoma and osteosarcoma (259). Proteases, such as ADAM-10, ADAM-17, and the membrane type matrix metalloproteinase 14, have been found to mediate MIC shedding through proteolytic activities (265–267). In an *in vitro* drug screen using a Federal Drug Administration-approved drug library, lomofungin, an antifungal drug, was found to strongly decrease ADAM17 activity in hepatocellular carcinoma cells and resulted in enhanced membrane bound MICA expression and inhibited soluble MICA production (261). Another ADAM17 inhibitor, INCB7839, was used to present HER2 cleavage and to treat patients with HER2-positive breast cancer in combination with trastuzumab and it is also in clinical trials to prevent CD20 cleavage in combination with rituximab for the treatment of diffuse large B-cell NHL (268). It would be interesting to investigate if these inhibitors prevent NKG2D ligands shedding and enhance NK-cell therapy for solid tumors. Applying neutralizing antibodies of soluble NKG2D ligands is another promising strategy to overcome immune suppressive effect of these cleaved ligands. Soluble MIC-specific mAb B10G5 was shown highly effective against primary prostate carcinoma and metastasis in the double transgenic TRAMP/MIC mouse model (262). B10G5 antibody therapy effectively induced regression of primary tumors and eliminated metastasis associated with enriched NK cell infiltration in the prostate tumor parenchyma (262). B10G5 therapy also remarkably restored NK cell pool in the periphery and the ability of NK cell homeostatic to self-renew as evidenced by bromodeoxyuridine uptake and markedly enhanced NK cell function, illustrated by increased production of IFNγ in response to mitogen stimulation and cytolytic ability against NKG2D ligand-positive target cells (262). These data conclude that targeting serum soluble MIC significantly restores NK cell homeostatic maintenance and function in MIC^+^ cancer host (262).

**Table 5:** Strategies to enhance NKG2D signaling for solid tumors.

**Agent**	**Function**	**Diseases**	**Report year**	**References**
NKG2D CAR NK	Enhance NKG2D expression on NK cells	Osteosarcoma, prostate carcinoma, rhabdomyosarcoma	2013	(196)
HDAC inhibitor Entinostat	Enhance NKG2D expression on NK cells	Colon carcinoma and osteosarcoma	2015	(259)
HDAC inhibitor Entinostat	Enhance NKG2D ligands MICA/B expression on tumor cells	Colon carcinoma and osteosarcoma	2015	(259)
HDAC inhibitor Sodium Valproate	Enhance NKG2D ligands MICA/B expression on tumor cells	Hepatoma cells	2005	(260)
Lomofungin	decrease ADAM17 activity and enhance MICA on tumor cells	Hepatocellular carcinoma	2018	(261)
B10G5	Soluble MIC neutralization monoclonal antibody	Primary prostate carcinoma and metastasis tumors	2015	(262)

## Enhancing NK Homing and Tumor Infiltration

Several studies have shown that NK cell homing and infiltration within tumors was associated with improved tumor regression and prognosis (7, 269). The inability of NK cells to migrate to the tumor site limits the clinical outcome of adoptive NK cell infusion in patients with solid tumors (270, 271). Strategies that increase NK homing and infiltration into tumors would be plausible to enhance NK antitumor efficacy and prevent resistance and relapse. The ability of NK cells to home and infiltrate into tumors largely depends on the chemokine receptors they express as well as the chemokines secreted by the tumor cells (272). Wennerberg et al. found that *ex vivo* expansion NK had significantly enhanced CXCR3 expression which resulted in increased migratory capacity toward CXCL10-producing RCC and melanoma tumor cells (273). Following adoptive transfer of these *ex vivo* expanded human NK cells, mice bearing CXCL10^+^ melanoma tumors had increased intratumoral infiltration of NK cells and a significantly prolonged survival compared with mice bearing CXCL10^−^ tumors (273). These data demonstrated the importance of CXCL10 in directing the migration and infiltration of CXCR3 human NK cells toward solid tumors (273). Prime the tumor microenvironment to secrete CXCL10 might be a good strategy to attract CXCR3 expression NK and to enhance the efficacy of NK cell-based therapy against solid tumors. Other efforts were made to genetically engineer NK cells with chemokine receptors to improve their migration toward the corresponding ligands on tumor cells surface. Various solid tumors, including RCC, secrete ligands for the chemokine receptor CXCR2 to promote angiogenesis, tumor growth and metastasis (274). Kremer et al. genetically engineered expanded human NK cells to express CXCR2 to improve their ability to specifically migrate along a tumor-derived chemokine gradient (271). CXCR2 expressing NK cells obtained increased adhesion properties and resulted in increased killing of target cells (271). Therefore, genetic engineering of *ex vivo* expanded NK cells to express chemokine receptor such as CXCR2 represents a novel strategy to improve anti-tumor effects following adoptive transfer of NK cells. A recent study connected the role of autophagy with CCL5-dependent NK cells infiltration in melanoma. Autophagy is a lysosomal degradation pathway for cells to self-digest their own components such as damaged organelles and misfolded proteins and such a degradation process provides nutrients to maintain cellular functions and allows survival of cancer cells under stress conditions (275, 276). Autophagy involves a Beclin-1 (BECN1)/class III phosphoinositide-3-kinase (PI3K) complex to initiate the formation of phagophore (275, 276). The previous studies from Baginska et al. demonstrated that targeting the autophagy gene *BECN1* prevented the degradation of NK-derived granzyme B, and therefore restored their susceptibility to NK cell-mediated killing and significantly inhibited tumor growth in syngeneic melanoma and breast mouse models (277). A recent study from Mgrditchian et al. found that when the autophagy process was blocked in tumor cells by inhibiting the expression of *BECN1*, the tumor cells produced an increased amount of CCL5 to attract functional NK cells to infiltrate into the melanoma tumor (276). Consequently, this led to a significant reduction in melanoma tumor size (276). These studies highlight the importance of integrating autophagy inhibitors as an innovative strategy in enhancing NK infiltration and killing.

## Targeting the Tumor Microenvironment and Blocking Transforming Growth Factor Beta (TGF-β) Pathway

It is well documented that the tumor microenvironment (TME) supports tumor growth, metastasis and suppress immune system (278). A major obstacle of ensuring high cytotoxic activity of NK cells is that these cells are surrounded by immunosuppressive cells and molecules in TME and must overcome the immunosuppressive properties from TME. One of immunosuppressive molecules is TGF-β1 (279). The increased TGF-β level was found in the plasma of advanced cancer patients such as breast cancer, ovarian cancer and neuroblastoma and correlated with worse event-free survival (280–282). Among three isoforms of TGF-β, TGF-β1 is the most abundant and widely studied isoform with 390 amino acids (283). This ligand binds to TGFβ receptor type I which results in its dimerization to TGFβ receptor type II and then phosphorylates SMAD2 and SMAD3 which complex with SMAD4 to modulate transcription of downstream genes (283, 284). TGF-β transmits biological signals to cells also through SMAD independent, alternative signaling pathways such as mitogen activated protein kinases, phosphoinositide 3′ kinase, and TNF receptor-associated factor6-TGF-β-activated kinase 1-p38/c-Jun N-terminal kinase (TRAF6-TAK1-p38/JNK) (283). TGF-β is produced by tumor cells themselves, Tregs, myeloid derived suppressor cells and other stromal cells in TME to downregulates the host immune response via driving the Th1/Th2 balance toward the Th2 immune phenotype, directly inhibiting anti-tumoral Th1-type responses and M1-type macrophages and promoting M2-type macrophages, suppressing cytotoxic CD8+ T-lymphocytes, NK, and dendritic cells functions, and stimulating CD4 + CD25+ T-regulatory cells (Treg) (285). TGF-β inhibits NK-cell proliferation and function in part by Treg cells which are known to produce high levels of TGF-β (286). One of the mechanisms by which TGF-β impairs NK cell function is by down-regulating the expression of NK activating receptors: NKp30 and NKG2D (287, 288). On the tumor side, TGF-β inhibits the transcription of the NKG2D ligands on tumor cells such as down regulation of MICA in the glioma cells, which reduced the recognition and killing by NKG2D expressing NK (289). TGF-β also represses development of NK cells from CD34+ progenitors and resulted in conversion of a minor fraction of CD56^bright^CD16^+^ cells found in peripheral blood into CD56^bright^CD16^−^ cells (290). TGF-β inhibits CD16-mediated human NK cell IFN-γ production and ADCC through SMAD3 (291). Further studies demonstrated that blockade of TGF-β signaling in NK cells caused the accumulation of NK cells that produce IFN-γ (292) and neutralization of TGF-β prevented NKG2D downregulation and also restored NK cell anti-tumor reactivity (293). RNA interference of TGF-beta1 and TGF-beta2 prevented the down-regulation of NKG2D on NK cells mediated by glioma cells and strongly enhanced MICA expression in the glioma cells and promotes their recognition and lysis by NK cells (289). These evidences support an immunosuppressive effect of TGF-β on NK cells and also provide a compelling rationale for blunting the inhibitory effect of it on NK cells as an anti-cancer therapy. Some approaches aiming at decreasing circulating TGF-β, blocking ligand-receptor interactions or inhibiting TGF-β signaling pathways to enhance NK based therapies are currently under investigation pre-clinically and clinically including TGF-β neutralizing antibody, TGF-β receptor I kinase inhibitors, SMAD3-Silenced NK Cells, NK cells engineered with a dominant negative receptor II for TGF-β, NK cells engineered to express a chimeric receptor with TGF-β type II receptor extracellular and transmembrane domains and the intracellular domain of NK cell-activating receptor NKG2D (Table 6).

**Table 6:** Summary of agents utilized to block TGF-β for solid tumors.

**Agent**	**Function**	**Diseases**	**Study stage**	**Clinical trial NCT #**	**Report year**
Fresolimumab (GC1008)	Human monoclonal antibody, neutrolize TGF-β	Advanced malignant melanoma and renal cell carcinoma	Clinical phase I	00356460	2014
Galunisertib (LY2157299)	Inhibitor of TGFβR1	Advanced solid tumors	Clinical phase I	01722825	2015
Galunisertib + Dinutuximab + aNK	Enhance ADCC of expanded NK cells	Neuroblastoma	Preclinical	N/A	2017
Galunisertib + aNK	Enhance cytotoxic function of expanded NK	Glioblastoma	Preclinical	N/A	2018
TGFBR2 knocking-down primary and expanded NK cells	TGF-β signaling pathway deficient	Brain tumor	Preclinical	N/A	2018
SMAD3 knocking-down NK-92 cells	TGF-β signaling pathway deficient	Hepatoma and melanoma	Preclinical	N/A	2018
DNRII CB NK	CB NK cells to express a dominant negative receptor II for TGF-β	Glioblastoma	Preclinical	N/A	2017
NK-92-TN (contains the TGF-β type II receptor extracellular, transmembrane domains and the intracellular domain of NK cell-activating receptor NKG2D (TN chimeric receptor)	Resistant to TGF-β-induced suppressive signaling, but did not downregulate NKG2D	hepatocellular carcinoma	Preclinical	N/A	2017

Fresolimumab (GC1008) is a high-affinity fully human mAb that neutralizes the active form of all the three isoforms of TGF-β (294). It was designed as an IgG4 isotype to minimize immune effector function. Fresolimumab has been assessed as a potential treatment for RCC and metastatic melanoma. The safety and antitumor activity of repeated doses of fresolimumab administered to patients with advanced malignant melanoma and RCC was evaluated in a Phase I study (NCT00356460) (294). Even the study was not designed to evaluate the effect of fresolimumab on NK cells but it showed acceptable safety and displayed encouraging antitumor activity (294). The results warrant further studies of it with NK therapy.

Galunisertib (LY2157299 monohydrate) is a small-molecule inhibitor of TGFβR1 that binds antagonistically to TGFβR1 to prevent the intracellular phosphorylation of SMAD2 and SMAD3 (295). Phase I studies have demonstrated that galunisertib had an acceptable tolerability and safety profile in patients with advanced solid tumors (296). Recently the preclinical studies from Tran et al. demonstrated that galunisertib combined with anti-GD2 antibody Dinutuximab augmented the anti-tumor cytotoxicity of activated NK (aNK) cells which were activated *ex vivo* with K562.mbIL21 artificial antigen presenting cells (297). Galunisertib suppressed SMAD2 phosphorylation and restored the expression of DNAX Accessory Molecule-1, NKp30, NKG2D and TNF-related apoptosis-inducing ligand death ligand expression on aNK cells and also significantly enhanced the release of perforin and granzyme A from aNK cells and the direct cytotoxicity and ADCC of aNK cells against neuroblastoma cells *in vitro* (297). The combination of galunisertib, aNK cells plus dinutuximab reduced tumor growth and increased survival of mice xenografted with two neuroblastoma cell lines or a patient-derived xenograft (297). In another study, galunisertib was shown to preserve the cytotoxic function of *ex vivo* expanded, highly activated NK cells and significantly improved eradication of liver metastases of colon cancer in mice treated with adoptive NK cells compared with mice receiving NK cells or TGF beta inhibition alone (298). Overall these studies demonstrate that the therapeutic efficacy of adoptive NK cell therapy clinically will be markedly enhanced by complementary approaches targeting TGF-beta signaling *in vivo*.

*Ex vivo* manipulating NK cells by novel strategies such as knocking-down TGF-β receptor 2 (TGFBR2) and SMAD3, expressing a dominant negative receptor II for TGF-β, or engineering with a TGF-β type II receptor based chimeric receptor to block the TGF-β signaling pathway are very attractive for adoptive NK therapy for solid tumors. Kararoudi et al. knocked down *TGFBR2* in human primary and expanded NK cells using the novel DNA-free Cas9 ribonucleoprotein complexes (299). *TGFBR2*-knockdown NK cells showed less sensitive to TGFβ (299). SMAD3 is a downstream factor in TGF-β signaling pathway and plays an essential role in TGF-β-mediated immune suppression, and in regulating transcriptional responses that are favorable to metastasis (300). *SMAD3* knocked-down NK-92 cells showed enhanced cancer killing activities and enhanced IFN-γ production *in vitro* and better anticancer effects than NK-92 empty vector control in non-obese diabetic severe combined immunodeficiency mice bearing human hepatoma (HepG2) or melanoma (A375) *in-vivo* (301). Yvon et al. engineered CB NK cells to express a dominant negative receptor II for TGF-β (DNRII) (302). These CB-derived DNRII-transduced NK cells were expanded to clinically relevant numbers, retained their secretion of interferon-γ, maintained both perforin and NKG2D/DNMA1 expression, and more importantly, retained their killing ability in the presence of TGF-β for glioblastoma cells (302). NK-92 cells were engineered to express a chimeric receptor which contains the TGF-β type II receptor extracellular, transmembrane domains, and the intracellular domain of NK cell-activating receptor NKG2D (TN chimeric receptor) by Wang et al. (303). These NK-92-TN cells were resistant to TGF-β-induced suppressive signaling, did not downregulate NKG2D (303). These modified NK-92 cells had higher killing capacity and IFN-γ production against carcinoma tumor cells compared with the control cells *in vitro* and in in a hepatocellular carcinoma xenograft tumor model (303). More interestingly, NK-92-TN cells were better chemo-attracted to the tumor cells expressing TGF-β and their cytotoxicity was further enhanced by TGF-β (303). The presence of these modified NK-92-TN cells significantly inhibited the differentiation of human naive CD4+ T cells to regulatory T cells (303). Overall, these engineered NK cells either with *SMAD*3 knock-down, expressing a dominant negative receptor II for TGF-β, or with a TGF-β type II receptor based CAR should have functional advantages over unmodified NK cells in the presence of TGF-β-secreting solid tumors and will be important therapeutic approaches for NK resistance in patients with solid tumors.

## Conclusion and Future Directions

**Figure 5:**
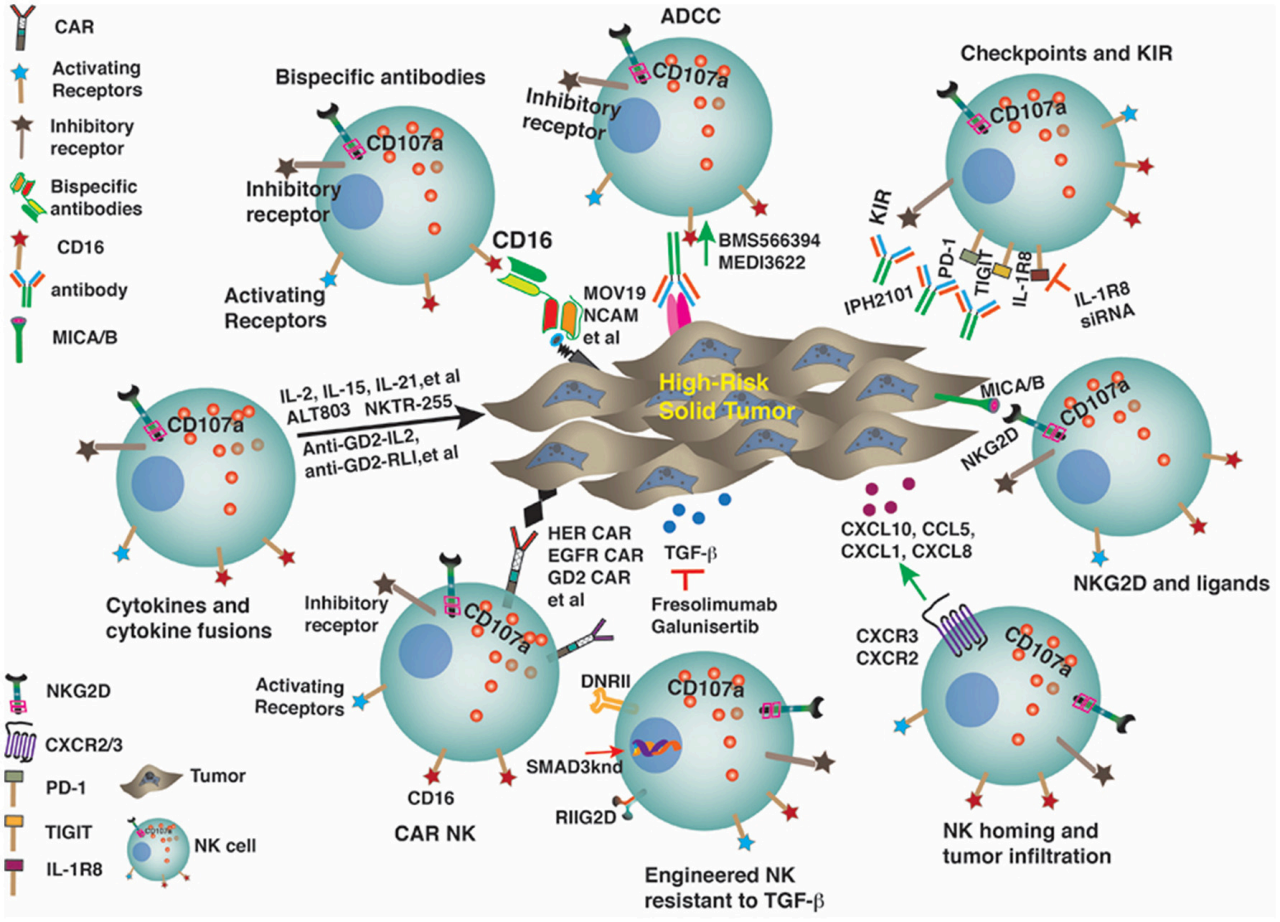
Strategies to overcome NK resistance in solid tumors. To enhance targeting specificity, NK cells have been engineered to express CAR such as anti-HER2 CAR, anti-EGFR CAR, anti-GD2 CAR et al. to target a specific antigen on tumor cell surface. NK cells can be activated by cytokines and cytokine fusion proteins such as IL-2, IL-15, IL-12, IL-18, IL-21, ALT-803 (an IL-15 superagonist), NKTR-255 (a polymer-engineered IL-15 molecule), anti-GD2-IL2, and anti-GD2-RLI fusions et al. Bispecific antibodies are novel molecules where two antigens can be targeted at the same time by combining the specificities of two antibodies. Bispecific antibodies can enhance NK cells targeting and killing. Preventing CD16 shedding and expressing high affinity CD16 on NK cells combined with novel engineered humanized antibodies will enhance NK mediated ADCC. The inhibitory roles of checkpoint proteins PD-1, TIGIT, IL-1R8, and KIR on NK cells are well documented. Blocking PD-1, TIGIT, and KIR with specific antibodies or knocking down IL-1R8 in NK cells unleash human NK-cell effector function. The membrane-bound ligands such as MICA/B binding to NKG2D play an important role in NK cell activation and tumor immune surveillance. Therapeutic strategies have focused on enhancing NKG2D expression and signaling on NK cells and enhancing the level of membrane-bound NKG2D ligand on tumor cells; and eliminating soluble NKG2D ligands. To enhance NK homing and tumor infiltration, NK cells can be enhanced to express chemokine receptors such as CXCR3, CXCR2 to be attracted to tumor cells that secret CXCL10, CXCL1, CXCL8, or CCL5. TGF-β plays an immunosuppressive effect of on NK cells. The approaches to block TGF-β and inhibit TGF-β pathway including TGF-β neutralizing antibody, TGF-β receptor I kinase inhibitors, SMAD3-Silenced (Smad3knd) NK Cells, NK cells engineered with a dominant negative receptor II for TGF-β (DNRII), NK cells engineered to express a chimeric receptor with TGF-β type II receptor extracellular and transmembrane domains and the intracellular domain of NK cell-activating receptor NKG2D (RIIG2D).

NK cell based applications are a promising alternative for immunotherapy of solid tumors. Improvements in understating of NK cell biology and function are driving the further development of NK cells based novel approaches to effectively target solid tumors. We have described the multiple strategies that have been investigated for improving the cytolytic properties of NK cells (Figure 5). In the future, combinations of these approaches need to be optimized to further enhance NK efficacy in targeting solid tumors. There is also a growing need to improve the current imaging modalities to monitor the accumulation and distribution of NK cells *in vivo* after systemic administration, which could serve as a potential surrogate for monitoring the tumor accumulation and anti-tumor response. Improvements in manufacturing and expansion techniques are desired in order to obtain a true universal “off-the-shelf” NK cell product that is GMP-compatible, lower in cost, has a longer half-life and possesses enhanced antitumor responses.

## Author Contributions

GN and YC reviewed the literatures, developed the designed for the paper, wrote the manuscript and contributed equally. MC reviewed and approved the final manuscript.

### Conflict of Interest Statement

The authors declare that the research was conducted in the absence of any commercial or financial relationships that could be construed as a potential conflict of interest.

## Abbreviations

ADCC: antibody-dependent cellular cytotoxicity
aNK: activated natural killer
CAR: chimeric antigen receptors
CEA: carcinoembryonic antigen
CB: cord blood
EGFR: epidermal growth factor receptor
EPCAM: epithelial cell adhesion molecule
FBP: folate binding protein
HER2: human epidermal growth factor receptor 2
HLA: human leucocyte antigen
HSCT: hematopoietic stem cell transplant
Hu: humanized
IgG: immunoglobulin g
IL: interleukin
IL1R: interleukin-1 receptor
INF-γ: interferon gamma
KIR: killer cell receptor
mAB: monoclonal antibody
MHC1: major histology antibody complex 1
MICA/B: MHC class I chain-related protein A/B
mRNA: messenger ribonucleic acid
NCAM: neural cell adhesion molecule
NK: natural killer cell
NKG2A: natural killer cell lectin-like receptor subfamily A
NKG2D: natural killer group protein 2 family member D
NKT: natural killer T cell
NSCLC: small lung cell carcinoma
PBMC: peripheral blood mononuclear cells
PD-1: programmed cell death 1
PDFGR: platelet-derived growth factor receptor
RCC: renal cell carcinoma
SABR: stereotactic ablative body radiotherapy
SCFv: single chain fragment variable region
SCT: stem cell transplantation
STAT: signal transducer and activator of transcription, TGF-β transforming growth factor beta
TME: tumor microenviroment
TNF: tumor necrosis factor
Tregs: regulatory T cells
Trikes: trispecific killer cell engagers.
